# IL-4 receptor dependent expansion of lung CD169^+^ macrophages in microfilaria-driven inflammation

**DOI:** 10.1371/journal.pntd.0007691

**Published:** 2019-08-30

**Authors:** Frédéric Fercoq, Estelle Remion, Stefan J. Frohberger, Nathaly Vallarino-Lhermitte, Achim Hoerauf, John Le Quesne, Frédéric Landmann, Marc P. Hübner, Leo M. Carlin, Coralie Martin

**Affiliations:** 1 Unité Molécules de Communication et Adaptation des Microorganismes (MCAM, UMR 7245), Sorbonne Université, Muséum national d’Histoire naturelle, CNRS; CP52, Paris, France; 2 Institute for Medical Microbiology, Immunology & Parasitology (IMMIP), University Hospital of Bonn, Bonn, Germany; 3 German Center for Infection Research (DZIF), partner site Bonn-Cologne, Bonn, Germany; 4 Leicester Cancer Research Centre, University of Leicester, Leicester, United Kingdom; 5 CRBM, University of Montpellier, CNRS, France; 6 CRUK Beatson Institute, Garscube Estate, Bearsden, Glasgow, United Kingdom; 7 Institute of Cancer Sciences, University of Glasgow, Glasgow, United Kingdom; Institut national de la recherche scientifique, CANADA

## Abstract

Lung disease is regularly reported in human filarial infections but the molecular pathogenesis of pulmonary filariasis is poorly understood. We used *Litomosoides sigmodontis*, a rodent filaria residing in the pleural cavity responsible for pleural inflammation, to model responses to human filarial infections and probe the mechanisms. Wild-type and Th2-deficient mice (*ΔdblGata1* and *Il-4receptor(r)a*^*-/-*^*/IL-5*^*-/-*^*)* were infected with *L*. *sigmodontis*. Survival and growth of adult filariae and prevalence and density of microfilariae were evaluated. Cells and cytokines in the pleural cavity and bronchoalveolar space were characterized by imaging, flow cytometry and ELISA. Inflammatory pathways were evaluated by transcriptomic microarrays and lungs were isolated and analyzed for histopathological signatures. 40% of WT mice were amicrofilaremic whereas almost all mutant mice display blood microfilaremia. Microfilariae induced pleural, bronchoalveolar and lung-tissue inflammation associated with an increase in bronchoalveolar eosinophils and perivascular macrophages, production of mucus, visceral pleura alterations and fibrosis. Inflammation and pathology were decreased in Th2-deficient mice. An IL-4R-dependent increase of CD169 was observed on pleural and bronchoalveolar macrophages in microfilaremic mice. CD169^+^ tissue-resident macrophages were identified in the lungs with specific localizations. Strikingly, CD169^+^ macrophages increased significantly in the perivascular area in microfilaremic mice. These data describe lung inflammation and pathology in chronic filariasis and emphasize the role of Th2 responses according to the presence of microfilariae. It is also the first report implicating CD169^+^ lung macrophages in response to a Nematode infection.

## Author summary

Filarial infections are tropical diseases caused by nematodes of the Onchocercidae family. Infections due to filariae regularly induce pulmonary manifestations, however lung pathogenesis of human filarial infections is poorly understood. *Litomosoides sigmodontis* is a rodent filaria living in the pleural cavity and responsible for pleural inflammation which is used to model responses in human filarial infections. Here, we analyse lung inflammation during the chronic phase of the infection, when female parasites release their offspring (microfilariae). 40% of mice do not present circulating microfilariae. We show that microfilariae enhance pleural cavity responses and induce pulmonary pathology (bronchoalveolar eosinophilia, perivascular cell infiltrates, mucus production and fibrosis of the visceral pleura). Numerous groups of tissue-resident macrophages are identified at homeostasis in the lungs which increase upon infection. Th2-deficient mice present higher parasite burden but lower inflammation and pathology. Our study provides new insight in filarial lung inflammation which may help to understand pathogenesis of human infections.

## Introduction

Human filarial infections are caused by nematodes of the Onchocercidae family. These parasites are transmitted by hematophagous arthropods and have a life cycle consisting of four larval stages (L1 to L4) with a moult occurring at the end of each larval stage, and an adult stage comprising separate males and females. Filarial infections cause several human diseases. Due to severe pathology that includes visual impairment and dermatitis for onchocerciasis, and lymphedema and hydrocele for lymphatic filariasis (LF) [1, 2],the World Health Organisation has marked both diseases for elimination. LF is caused by *Wuchereria bancrofti*, *Brugia malayi* and *B*. *timori*, mansonellosis by *Mansonella perstans* and zoonotic filariasis is mainly caused by *Dirofilaria immitis* a species that normally infects non-human animal hosts. Pulmonary manifestations of filarial infections are regularly reported in these human diseases [3–5].

Although infections with *M*. *perstans* are often considered asymptomatic, pleural effusions, pulmonary hypertension and peribronchic infiltrates have been described [5–7]. Tropical pulmonary eosinophilia (TPE) is a rare but documented manifestation of LF. The current concept of the pathogenesis of TPE suggests that it begins with lung parenchymal inflammation in individuals highly immunologically sensitized to filarial parasites. The offspring, microfilariae (Mf; L1), released from adult worms living in lymphatics are cleared in the pulmonary circulation, degenerate and release their antigenic constituents which trigger local inflammation [4, 8] leading to an asthma-like syndrome with eosinophilia, peribronchial infiltrates and fibrosis [4, 9, 10]. In human pulmonary dirofilariasis the parasites accumulate in the pulmonary artery where they embolize, ultimately leading to the formation of a pulmonary nodule or coin lesion on chest x-rays [11]. Importantly, and independent of the filarial species causing the infection, the molecular pathogenesis of pulmonary filariasis is poorly understood.

*Litomosoides sigmodontis* is a rodent filaria which is used to model the host response in human filarial infections [12]. Infective larvae (L3) migrate from the skin to the pleural cavity (PC) within eight days [13], where they remain for the duration of infection. In BALB/c mice, parasites mature and mate, and in about 60% of mice they release Mf that circulate in the bloodstream from approximately day 55 post infection (p.i.). In approximately 40% of mice, Mf are not observed. However, LF or mansonellosis patients are also often amicrofilaremic [2, 5, 14]. Independently of their Mf status, cotton rats and jirds exhibit some pulmonary lesions in the patent phase of *L*. *sigmodontis* infection between 60 and 100 days p.i. [15, 16]. Pleural cell infiltrates have been characterized through the filarial development [13, 17, 18], and more recently, inflammation of the visceral pleura was observed in patent BALB/c mice [19].

The aim of this study was to provide new insight into Mf-driven lung pathology during filariasis.

## Material and methods

### Ethics

All experimental procedures were carried out in accordance with the EU Directive 2010/63/UE and the relevant national legislation, namely the French “Decret No. 2013±118, 1^er^ fevrier 2013, Ministère de l'Agriculture, de l'Agroalimentaire et de la Foret”. Protocols were approved by the ethical committee of the Museum National d'Histoire Naturelle (Comité Cuvier, Licence: 68–002) and by the Direction départementale de la cohésion sociale et de la Protection des populations° (DDCSPP) (No. C75-05-15).

### Rodents and infestation protocol

Maintenance of the filaria *L*. *sigmodontis* Chandler, 1931 and recovery of infective larvae (L3) from the mite vector, *Ornithonyssus bacoti*, were carried out as previously described [20]. Six weeks-old female BALB/c OlaHSD mice were obtained from Envigo; 8-week-old jirds were purchased from Janvier. Genetically modified BALB/c mice were provided by Dr Hübner (Bonn). These included six to ten weeks-old homozygous *ΔdblGata1* female BALB/c mice, which present a deletion of a high-affinity GATA-binding site in the GATA-1 promoter leading to selective a loss of the eosinophil lineage [21]; six to ten weeks-old *Il-4ra*^*-/-*^*/Il-5*^*-/-*^ female BALB/c mice, which are deficient for the α chain of the IL-4 receptor (IL4-Ra) and thus lacking IL-4/IL-13 signaling and for IL-5, leading to an absence of alternative activation of macrophages and an impaired maturation and recruitment of eosinophils, and a substantial microfilaremia when infected with *L*. *sigmodontis* [15, 16, 19, 22, 23]. All animals were maintained in the MNHN animal facilities on a 12-hours light/dark cycle. Mice were inoculated subcutaneously with a single dose of 40L3. Analyses were performed in the patent phase at D70 p.i.

### Dissection of mice, pleural and bronchoalveolar cells isolation, and filarial recovery

At necropsy, filariae (F) were recovered with pleural cells by lavaging the pleural cavity with 10 ml cold PBS as described in [13]. Bronchoalveolar cells were recovered by flushing of the bronchoalveolar space with 10 ml PBS as described in [13]. The first ml of pleural wash and the first ml of bronchoalveolar lavage were stored for further immunological analysis. Pleural cells and bronchoalveolar cells were resuspended in 1 ml of PBS+2% FCS. Red blood cells were removed by hypotonic lysis and pleural cells and bronchoalveolar cells were counted. Filariae were harvested, counted, fixed in 70% ethanol and analysed by light microscopy (Olympus BX63 microscope, DP72 camera). They were measured using CellSens Dimension 1.9 software. The recovery rate of filariae, expressed as 100 x number of worms recovered/number of larvae inoculated (F/L3) was established.

### Uterine content analysis of female filariae

Female filariae from WT Mf^neg^, WT Mf^pos^, *ΔdblGata1* Mf^pos^ and *Il-4ra*^*-/-*^*/Il-5*^*-/-*^ Mf^pos^ BALB/c mice were incubated with DAPI (1:1000) for 1h and mounted in Fluoroshield Mounting Medium with DAPI (Abcam). Images from the proximal portion of the uteri (close to ovojector) were captured with an inverted laser scanning confocal microscope (SP5-SMD; Leica Microsystems) with a z-stack of 20μm. The images were processed and analysed using IMARIS (Bitplane) to measure the proportion of microfilariae over the whole developmental stages present in the proximal uteri portion.

### Microfilaremia and purification of microfilariae

Peripheral blood microfilaremia (number of microfilariae) was determined at day 60 and 70 p.i. on a 10μl Giemsa stained blood drop. Cardiac blood microfilaremia was determined at day 70 p.i.

Microfilariae (Mfs) were isolated as described in [24]. Briefly, blood from microfilaremic jirds was collected and the microfilariae were purified using a sucrose/Percoll density gradient, resuspended in 1mL PBS and counted.

### Cell analysis

Pleural and Bronchoalveolar cells were preincubated with murine Fc block CD16/CD32 and then stained with the following rat anti-mouse antibodies: anti-F4/80-APC (1:200; eBioscience, clone BM8), anti-SiglecF-PE (1:200, BD Bioscience, clone E50-2440), anti-Ly6G-V450 (1:200, eBioscience, clone 1A8), anti-CD4-PE (1:200; BD Bioscience, clone RM4-5) and anti-CD19-APC (1:200; BD Bioscience, clone 1D3). Macrophages were further analysed using anti-CD169-FITC (1:200, Biolegend, clone 3D6.112) and CD206-PE-Cy7 (1:200, Biolegend clone C068C2). Fluorescence Minus One (FMO) controls were used for each group with a pool of cells of all groups. The samples were run on a FACSVerse flow cytometer (BD Biosciences) and analysed using FACSuite software. Doublets and debris were excluded. CD169 and CD206 expression is expressed as mean fluorescence intensity (MFI) normalized by FMO (MFI-FMO).

### ELISA cytokine assays

Pleural wash (diluted 1:4) or bronchoalveolar (diluted 1:4) fluids collected from individual mice were assayed for cytokine content by enzyme-linked immunosorbent assay (ELISA) in duplicate. These assays were performed according to the manufacturers’ recommendations, using the following kits, IFN-γ, CCL2, IL-4, IL-6 (eBiosciences SAS, France), CCL11 (Peprotech, France) and CXCL9 (R&D, UK). Results are shown as pg/mL. Detection limits were 4 pg/ml for IL-4 and IL-6, 15 pg/ml for INF-γ, CCL2 and CXCL9 and 30 pg/ml for CCL11.

### Lung analysis by Scanning Electron Microscopy (SEM)

Lungs from naive and *L*. *sigmodontis* 70-day-infected WT mice and 6-months-infected jirds were removed from the chest, placed in a petri dish containing PBS and cut in 3-4mm thick sections. Sections were fixed with 2.5% glutaraldehyde, dehydrated with increasing concentrations of ethanol (from 50 to 100%), and dried with hexamethyldisilane (HMDS). Samples were then fixed on metal supports using double-sided carbon tape and metallized by sputtering gold (Jeol FJC-1200 metallizer). Observations of the visceral pleura were made with a Hitachi SU3500 SEM (MNHN Technical Electron Microscopy Platform).

### Lung histology and immunohistology on thin sections

Lungs from naïve and *L*. *sigmodontis* infected WT, *ΔdblGata1* and *Il-4ra*^*-/-*^*/Il-5*^*-/-*^ BALB/c mice (n = 7–15 per group) were inflated with and fixed in 4% formalin, dehydrated in 70% to 100% ethanol baths, and then placed in toluene before paraffin embedding. Four-micron-thick serial sections were prepared and various stainings were performed: 1) Picrosirius red (Bio optica, Italy) and Masson's trichrome (Sigma-Aldrich) to visualize collagen fibers according to the manufacturers' recommendations; 2) Alcian Blue/Periodic Acid Schiff (AB-PAS) staining to visualize mucus producing cells using the following protocol: http://www.ihcworld.com/_protocols/special_stains/alcian_blue_pas_ellis.htm; 3) a cytokeratin immunostaining to visualize mesothelial cells; briefly antigens retrieval was performed using a solution of Proteinase K (10 μg/ml) in Tris-EDTA buffer, then peroxidases and endogenous alkaline phosphatases were blocked by adding DualEndogenous Enzyme Block (Dako, France). Sections were incubated with the mouse anti-human cytokeratins monoclonal Ab (1:50, clone AE1/AE3, Dako). Binding of the antibodies was detected by HRP linked universal secondary antibody (DAKO) and AEC substrate (DAKO). The sections were counterstained with a Mayer Hematoxylin solution. Lung sections were analysed by light microscopy (Olympus BX63 microscope, DP72 camera) using the cell Sens Dimension 1.9 software. Full lobe sections were imaged by mosaic imaging and pleural pathology (100 x length of pathologic pleura / total perimeter) and bronchial inflammation (100 x nb of AB-PAS positive bronchus sections / total nb of bronchus sections) were measured. For each parameter, 2–3 lung sections were analysed.

### Static Precision Cut Lung Slice (PCLS) imaging

Lungs from naive and *L*. *sigmodontis* infected WT, *ΔdblGata1* and *Il-4ra*^*-/-*^*/Il-5*^*-/-*^ BALB/c mice (n = 4–11 per group) were prepared for confocal microscopic analysis. PCLS were preoduced as previously described [13]. Briefly, after removal of filariae, lungs were inflated with 2% low melting point agarose (Sigma-Aldrich) in PBS (40°C, pH 7.4) and covered with ice for 5 min. Lungs were removed from the pleural cavity, rinsed in PBS, fixed in PBS/PFA 4% for 2h at 4°C and stored in cold PBS/BSA1%/Azide 0.05%. The left lung was isolated and cut with a vibrating microtome (Campden 5100mz) into 300-μm slices. Lung slices were stained with the following cocktail at room temperature: first a hamster anti-mouse CD31 antibody (2H8, Life Technologies, 1:200) was applied for 3h; secondly Alexa Fluor 488 goat anti-hamster (1:200, polyclonal, Jackson), Alexa Fluor 594 rat anti-mouse CD68 (1:200, clone FA-11, Biolegend, company), Alexa Fluor 647 rat anti-mouse CD169 (1:200, clone 3D6.112, Biolegend), and DAPI (1:1500) were added for 1h. All antibodies were diluted in PBS/NGS 10%/BSA1%/TX-100 0.3%/Azide 0.05%. Several PBS washing steps were performed before and after a 2 min 4% PFA fixation and slices were transferred to glass slides, covered with buffered Mowiol 4–88, pH 8.5 (Sigma-Aldrich) then coverslipped. Areas of about 1mm x 1mm side by 100μm deep containing perivascular spaces or visceral pleura were acquired with a confocal microscope (Airyscan 880; Zeiss). At least 2 images of PVS and 1 of visceral pleura were obtained for each lung.

Images were analysed using the IMARIS software (Bitplane). The different planes (z) were stacked to obtain a three-dimensional reconstruction of the different fluorescence signals. Perivascular space volume was measured over the 50μ z-stack (in mm^3^) using the software tools and the number of cells present in the space (DAPI^+^ and DAPI^+^CD68^+^CD169^+^) was counted to determine cell concentration in the different PVS images (number cells/mm^3^).

### Lung Mf detection and quantification

Lung DNA was extracted to allow the detection and quantification of pulmonary microfilariae. The protocol was adapted from Bouchery *et al*. 2012. First, an 8 point standard curve was generated using lungs from naive mice to which a known number of Mf (from 0 to 1.000.000) was added before DNA extraction (see above for Mf purification).

Lungs (+/- Mf) were homogenized in a fixed volume of PBS (500 μl) using a Tissue Lyser II (Qiagen). 100μL of homogenate solution was used for genomic DNA extraction (QIAamp DNA Mini Kit, QIAGEN, Germany) according to the manufacturer’s protocol and finally eluted in 150 μl of sterile water. A real-time PCR was performed with the DNA Master Plus SYBR Green Kit (Roche Diagnostics, Meylan, France) in a LightCycler (Roche Diagnostics) with an initial incubation of ten minutes at 95°C, 40 amplification cycles of ten seconds at 95 °C, of five seconds at 60 °C, and of ten seconds at 72 °C, during which the fluorescence data were collected. The 10 μl reaction mixture contained 1X DNA Master Plus SYBR Green, 4 μM of each primer, and 4 μl of template. Filarial DNA and murine DNA were detected by targeting the actin of *L*. *sigmodontis* (*L*.*s* Actin 5'-ATCCAAGCTGTCCTGTCTCT-3’; 5'-TGAGAATTGATTTGAGCTAATG-3’) and the actin of *Mus musculus* (*M*.*m* Actin 5'-ATTGCTGACAGGATGCAGAAG-3’; 5'-AGTCCGCCTAGAAGCACTTG-3’) respectively. For each sample, the ratio (R) of signal (CT) from filarial actin and murine actin was performed to normalize the results as R = CT (*L*.*s* Actin) / CT (*M*.*m* Actin).

The number of microfilariae in the lung of infected WT, *ΔdblGata1* and *Il-4ra*^*-/-*^*/Il-5*^*-/-*^ BALB/c mice (n = 9–12 per group, 1 for *ΔdblGata1*) was then extrapolated using this ratio and the standard curve.

### Lung cytokine expression analysis

Screening of inflammatory lung environment was performed with a qRT-PCR array (Mouse Cytokines & Chemokines RT^2^ Profiler PCR Array, Qiagen, Germany) according to manufacturer’s instructions. RNA extraction was performed on RNeasy Midi kit colums (Qiagen). The quantity and quality of the RNAs was verified with a spectrophotometer (Nanodrop2000, Thermo Scientific) and an Agilent 2100 bioanalyzer. The complementary DNAs (cDNAs) were produced with the First-strand cDNA kit (Qiagen). A pool of cDNA from naive mice lungs (n = 8) was compared with one from D70 p.i. infected mice (n = 8) by profiling 84 cytokine-related genes simultaneously. The real-time PCR cycling program (7300 real-time PCR System, Applied biosystem) was run and data were processed and displayed using the online RT^2^ Profiler PCR Array Data analysis 3.5 software (Qiagen). Gene expression was normalized with 4 housekeeping genes (*Actb*, *Gapdh*, *Gusb*, *Hsp90ab1*). Transcripts with a fold change *>*2 were selected (raw data are available on GEO https://www.ncbi.nlm.nih.gov/geo/query/acc.cgi?acc=GSE115596).

Array results were validated by performing qRT-PCR on all individual samples (n = 8/group) for the highly upregulated genes *Cxcl9*, *Ccl2* and *Il1-3* with the following couples of primers: *Cxcl9*, 5’-CCATGAAGTCCGCTGTTCTTTTCC-3’; 5’-TGGGGCAAACTGTTTGAGGTCT-3’; *Ccl2*, 5’-ACTGCATCTGCCCTAAGGTCTTCA-3’; 5’-TAAGGCATCACAGTCCGAGTCACA-3’; *Il-13*, 5’-GGATATTGCATGGCCTCTGTAACC-3’; 5’-GTGGCGAAACAGTTGCTTTGTG-3’. A DNA Master Plus SYBR Green Kit (Roche Diagnostics, France) was used in a LightCycler 2.0 (Roche Diagnostics, France) with an initial incubation of 10 min at 95°C, 40 amplification cycles of ten seconds at 95°C, of 8 seconds at 60°C, and of 10 sec at 72°C, during which the fluorescence data were collected. The 10 μL reaction mixture contained 1X DNA MasterPlus SYBR Green (QIAGEN, France), 0.5 μM of each primer, and 5 μL of template. Gene expression was then determined relative to β-actin and GAPDH using the 2-ΔΔCT method.

Transcriptional data were evaluated using Ingenuity Pathway Analysis (IPA, Systems Inc., USA) and the activation of biological functions occurring in the tissue was predicted (IPA Core Analysis).

### Statistical analyses

Data analyses were performed with Prism 5.0 software (GraphPad Inc.). The choice of statistical tests was based on sample size and normality (Shapiro-Wilk test) examined prior to further analysis. Data from independent experiments were pooled when possible. When normality was established, results were analysed by one-way ANOVA test in order to determine the effect of one factor, followed by a Bonferroni's multiple comparisons post-test; otherwise non-parametric Kruskal Wallis tests followed by a Dunn's multiple comparisons post-test were used. Correlations between two datasets were analysed using the Pearson test. In all figures, the mean value is visually depicted. P values correlate with symbols as follows: ns = not significant, p > 0.05, * p ≤ 0.05, ** p ≤ 0.01, *** p ≤ 0.001. Mice were allocated randomly into experimental groups after matching for age. Specific numbers of animals can be found in corresponding figure legends.

## Results

### The lungs of filariae-infected rodents show overlying lesions and chronic inflammation of visceral pleura

**Fig 1:**
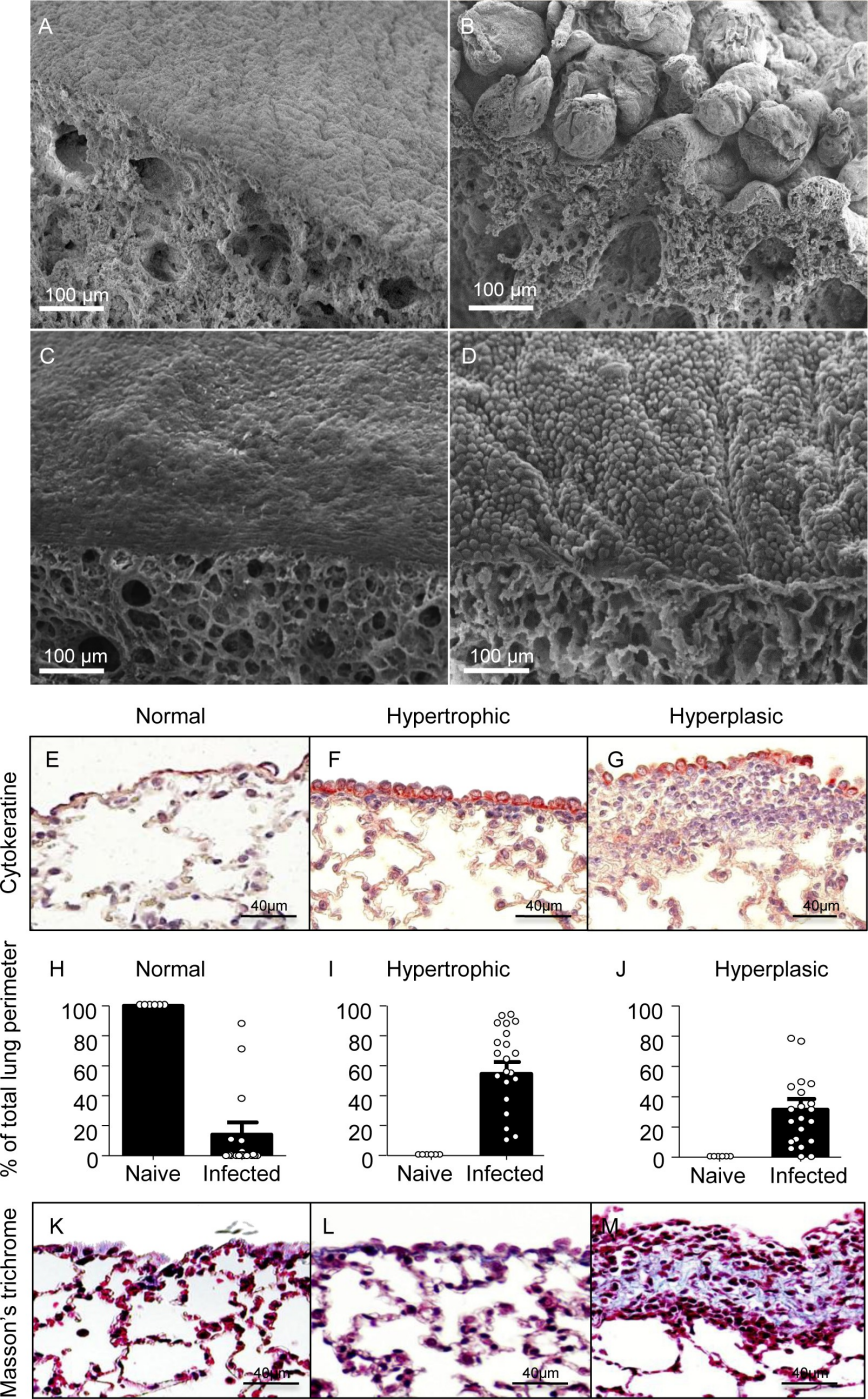
Visceral pleura pathology and fibrosis in rodent lungs during chronic filarial infection. (**A-B**) Jirds infected with *L*. *sigmodontis* were euthanized 6 months p.i., and lungs were recovered and prepared for scanning electron microscopy. (**A**) Normal lung visceral pleura (with pavimentous mesothelium) in a naive jird; (**B**) pathological bullous visceral pleura in an infected jird. (**C-M**) BALB/c mice infected with *L*. *sigmodontis* were euthanized 70 days p.i. and lungs were recovered. (**C-D**) Lungs were prepared for scanning electron microscopy. (**C**) Normal pleura in a naive 16-week-old BALB/c mouse; (**D**) pathological pleura with cuboidal mesothelium in an infected mouse. (**E-M**) lungs were paraffin-embedded and 4μm-section were prepared (**F-G**) Mesothelial cells were stained by anti-cytokeratine antibodies (red) revealing (**F**) hypertrophic mesothelial cells and (**G**) hyperplasic area below the mesothelial cells. (**H-J**) Quantification of hypertrophic (**I**) and hyperplasic (**J**) areas on the visceral pleura. (**K-M**) Staining for collagen (in blue) using Masson’s trichrome showing normal mesothelium (**K**), hypertrophic mesothelium (**L**) and accumulation of collagen in hyperplasic visceral pleura (**M**). Results are expressed as mean ± SEM (pool of 3 independent experiments) of n = 10 naïve mice, n = 22 infected mice.

The life cycle of the filaria *L*. *sigmodontis* is maintained in jirds (S1 Fig), a rodent similarly permissive to the cotton rat, its natural host. Upon opening the pleural cavity, polyps on the lungs are regularly observed. Scanning electron microscopy of the lungs of infected jirds, six months p.i. revealed marked pathology of the visceral pleura with a bullous/hairy appearance, composed of nodule-like structures (Fig 1A and 1B). To a much lesser extent, cuboidal ‘swollen’ mesothelial cells were observed on visceral pleura of BALB/c mice (Fig 1C and 1D). However, histology of infected mice revealed important modifications of the visceral pleura. Normal visceral pleura is composed of a single layer of pavimentous mesothelial cells expressing cytokeratine (Fig 1E). In infected BALB/c mice, mesothelial cell hypertrophy (increased cell volume) and hyperplasia of the visceral pleura (increased cell number) were observed (Fig 1F and 1G). Quantification (Fig 1H–1J) showed that all infected mice had a large portion of visceral pleura with hypertrophic mesothelial cells and hyperplasic areas (about 60% and 35% of the total perimeter of lung sections respectively (Fig 1I and 1J). Hyperplasic areas contained a dense mesh of collagen fibers, a signature of localized fibrosis, in contrast to normal and hypertrophic areas (Fig 1K–1M).

### Chronic filariasis triggers specific inflammatory response pathways in the lung

**Fig 2:**
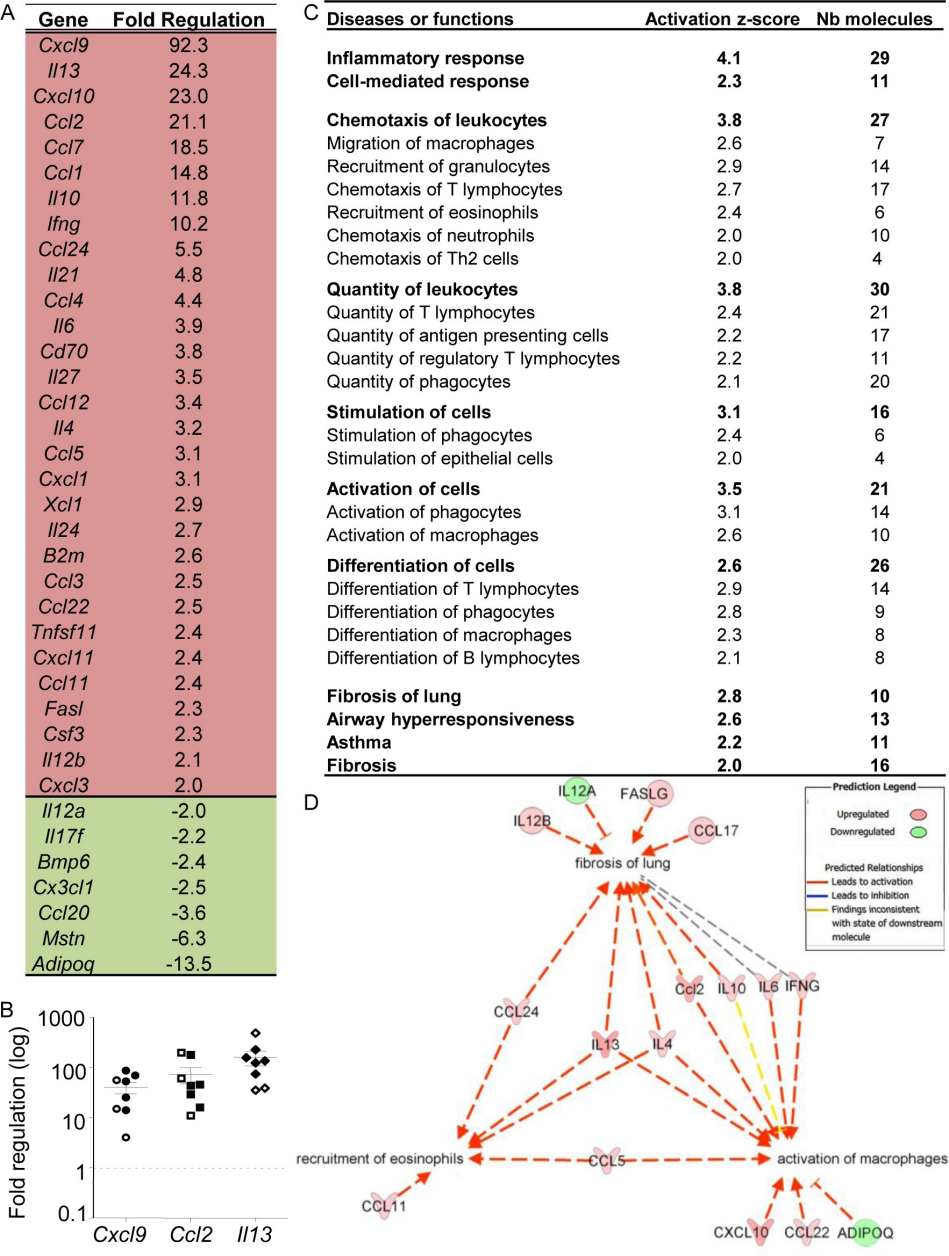
Analysis of cytokine gene expression changes in lungs during filarial infection. Lungs were recovered from *L*. *sigmodontis* infected BALB/c mice at 70 days p.i. and processed for gene expression profiling of cytokines/chemokines. (**A**) Analysis of differentially expressed genes; Fold change cut-off >2; cDNA pools from 8 naive and 8 infested mice; (**B**) individual qRT-PCR analysis for *Cxcl9*, *Ccl2* and *I113* transcripts; results are expressed as mean ± SEM (n = 8 naive and 8 infested mice). Plain symbols indicate microfilaremic mice, open symbols correspond to amicrofilaremic mice (**C**) Pathway analysis of selected gene-related functions reveals a strong inflammatory reaction in infected lungs. Gene expression profiles were analyzed with Ingenuity Pathway Analysis (IPA) to predict the functional consequences of the infection. The first column indicates the main diseases or functions predicted to be activated (the full list is presented on S1 Table); the second column shows the Activation z-score calculated by the software (activation if z-score≥2) and the third one indicates the number of molecules from the array involved in the disease or function. (**D**) A molecular network linking macrophage activation, eosinophil recruitment and pulmonary fibrosis was generated using the transcript profiles. Edges and nodes are color-coded based on the predicted relationships as indicated in the Prediction Legend.

To characterize lung inflammation in filariae-infected rodents, an analysis of cytokine and chemokine transcripts was performed on BALB/c lungs at 70 days p.i. The expression of 84 cytokine/chemokine-coding genes was analyzed and an upregulation of the expression of 30 genes and the downregulation of 7 genes was observed in infected mice compared to naïve mice, with some mixed Th1/Th2 signatures (Fig 2A). Among them, the expression of the Th1-related cytokines *Cxcl9*, *Cxcl10* and *Ifn-ɣ* were highly increased, as well as the prototypical Th2 cytokines *Il-13* and *Il-4*. *Il-5* expression was not modulated at this timepoint. Results were validated by individual qRT-PCR for the highly upregulated genes *Cxcl9*, *Ccl2* and *Il-13* (Fig 2B). Microfilaremic and amicrofilaremic BALB/c mice are similar.

*In silico* analysis of the results indicated robust lung inflammation, associated with the recruitment and activation of myeloid cells (especially macrophages and eosinophils) and lymphocytes (B cells, Th2 cells and Tregs) (Fig 2C and S1 Table). Furthermore, the development of Th2-associated lung pathology, such as fibrosis, asthma or airway hyper-responsiveness was also predicted (Fig 2C). A molecular network linking macrophage activation, eosinophil recruitment and pulmonary fibrosis was generated using the transcriptional profiles, highlighting a central role of IL-13 and IL-4 in pathogenesis and cell recruitment/activation (Fig 2D).

### The fertility and survival of filariae are dependent on a Th2 background

To understand the role of Th2 cytokines in parasite outcome, two genetically modified strains of mice were compared to BALB/c wild type (WT) mice: 1) the *ΔdblGata1* BALB/c mice, which lack the eosinophil lineage [21]; 2) the *Il-4ra*^*-/-*^*/Il-5*^*-/-*^ BALB/c mice, characterized by an absence of alternative activation of macrophages and impaired maturation and recruitment of eosinophils [15, 16, 19, 22].

**Fig 3:**
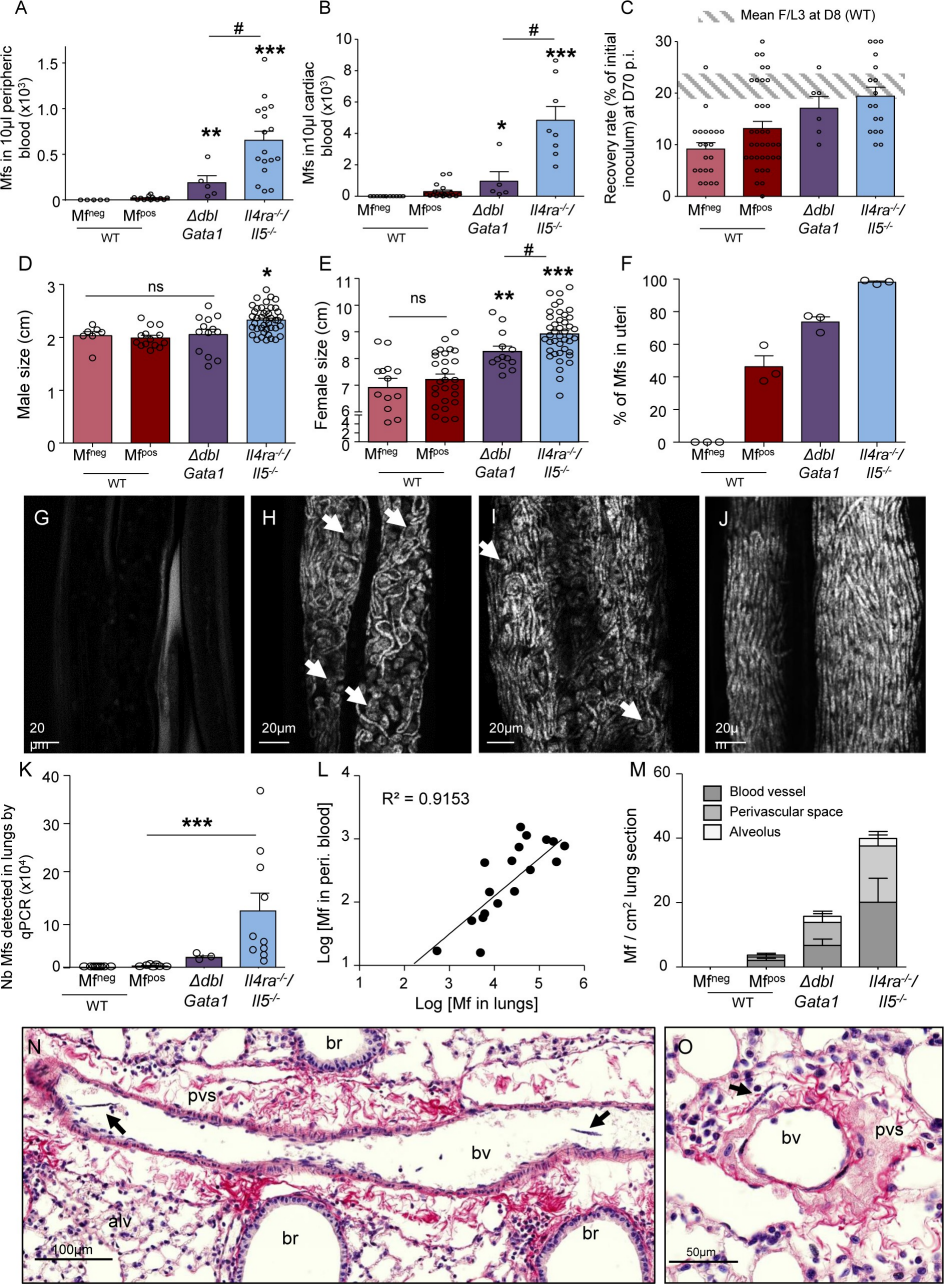
Filarial burden and growth are dependent on eosinophils, IL-5 and IL-4R. *L*. *sigmodontis* infected wild type (WT), *ΔdblGata1* and *Il-4ra*^*-/-*^*/Il-5*^*-/-*^ BALB/c mice were compared at 70 days p.i. for their microfilarial load in (**A**) peripheric blood or (**B**) cardiac blood (Mf are 10-fold more concentrated in cardiac blood than peripheral blood, allowing us to characterize Mfpos mice that would have been misdiagnosed using only peripheral blood). Results are expressed as mean ± SEM (n = 11 WT Mf^neg^, n = 19 WT Mf^pos^, n = 5 *ΔdblGata1* Mf^pos^, n = 8 *Il-4ra*^*-/-*^*/Il-5*^*-/-*^ Mf^pos^). Kruskal-Wallis followed by a Dunn's Multiple Comparison Test: *p<0.05. **p<0.01, ***p<0.001 represent differences between Mf^pos^ mice; #p<0.05 represent differences between mutant mice. (**C**) the recovery rate (F/L3) of adult worms in the pleural cavity; results are expressed as mean ± SEM. n = 21 WT Mf^neg^, n = 35 WT Mf^pos^, n = 6 *ΔdblGata1* Mf^pos^, n = 17 *Il-4ra*^*-/-*^*/Il-5*^*-/-*^ Mf^pos^. (**D**) the size of male adult filariae (n = 8–43 males, pool of 2 independent experiments) and (**E**) the size of female adult filariae (n = 13–42 females, pool of 2 independent experiments). Results are expressed as mean ± SEM. One-way Anova followed by a Bonferroni's Multiple Comparison Test: *p<0.05. **p<0.01, ***p<0.001 represent differences between Mf^neg^ and Mf^pos^ mice; #p<0.05 represent differences between mutant mice. (**F-J**) Female parasites were stained with DAPI and uterine content was observed by confocal microscopy. (**F**) Proportion of Mfs *versus* all developmental stages in the proximal uteri portion (close to ovojector) in female filariae from the different groups of mice. Results are expressed as mean ± SEM. n = 3 filariae per group; 3 images per filaria were analyzed. (**G-J**) Representative maximum intensity projection from confocal z-stack (z = 10μm) of the proximal uteri from (**G**) a female filaria from a WT Mf^neg^ mouse showing empty uteri; (**H**) a female filaria from a WT Mf^pos^ mouse showing uteri filled with Mfs and aborted embryos (white arrows); (**I**) a female filaria from a *ΔdblGata1* Mf^pos^ mouse. Aborted embryos are shown by white arrows; (**J**) a female filaria from a *Il-4ra*^*-/-*^*/Il-5*^*-/-*^ Mf^pos^ mouse showing uteri filled with Mfs. (**K-O**) Microfilariae have also been analysed in lung tissue: (**K**) Number of Mfs in lungs was estimated by qPCR; results are expressed as mean ± SEM. n = 9 WT Mf^neg^, n = 9 WT Mf^pos^, n = 3 *ΔdblGata1* Mf^pos^, n = 12 *Il-4ra*^*-/-*^*/Il-5*^*-/-*^ Mf^pos^; Kruskal-Wallis followed by a Dunns multiple comparison test: ***p<0.001, difference between Mf^pos^ mice. (**L**) Correlation test (Pearson) between the number of Mf in peripheral blood and the number of Mf detected by qPCR; r^2^ = 0,9086. (**M-O**) Lung sections were analyzed to localize and quantify Mf; peri. = peripheric (**M**) Distribution of Mf in the lungs; results are expressed as mean ± SEM. n = 6 WT Mf^neg^, n = 6 WT Mf^pos^, n = 3 *ΔdblGata1* Mf^pos^, n = 12 *Il-4ra*^*-/-*^*/Il-5*^*-/-*^ Mf^pos^. (**N-O**) Hematoxylin and Sirius red staining (collagen in red) of lung sections showing Mf (black arrows) in (**N**) a blood vessel and in (**O**) a collagenous perivascular space (pvs). br = bronchus; bv = blood vessel; alv = alveoli.

Mf were counted in peripheral and cardiac blood of the WT and mutant mice. This revealed striking differences between the genotypes (Fig 3A and 3B): 63% of WT mice show circulating Mf (Mf^pos^) but 83% of *ΔdblGata1* and 100% of *Il-4ra*^*-/-*^*/Il-5*^*-/-*^ were Mf^pos^ [23]. Moreover, Mf in peripheral blood and heart were more numerous in *Il-4ra*^*-/-*^*/Il-5*^*-/-*^ than in WT Mf^pos^ mice, and *ΔdblGata1* showed an intermediate phenotype (Fig 3A and 3B).

In WT mice, L3 migration is complete by D8 p.i. and survival of worms is stable until 2 months p.i. after which worm-burden decreases at D70 p.i. compared with D8 p.i. ([13] and S1 Fig). In contrast, worm burden remained stable in both groups of mutant mice indicating extended worm survival (Fig 3C). Filariae were longer in mutant mice (males only in *Il-4ra*^*-/-*^*/Il-5*^*-/-*^ mice (Fig 3D) and females in both mutant groups, longest in *Il-4ra*^*-/-*^*/Il-5*^*-/-*^ (Fig 3E)). WT Mf^pos^ and Mf^neg^ mice did not present adult worm size differences. Interestingly, the uterine content of female worms themselves was different depending on the host genotype. Uteri of female parasites from WT Mf^neg^ were empty of microfilariae (Fig 3F and 3G) while the proximal portion of uteri, close to the ovojector, of worms in Mf^pos^ mice (WT, *ΔdblGata1* and *Il-4ra*^*-/-*^*/Il-5*^*-/-*^) contained a mix of viable Mf with embryos that did not undergo morphogenesis (Fig 3H–3J). The number of aborted embryos was high in parasites from WT mice (half of uterine content) and almost null in those from *Il-4ra*^*-/-*^*/Il-5*^*-/-*^ mice; with *ΔdblGata1* showing an intermediate phenotype.

Mf presence was also estimated in lungs by qPCR and similar results to blood were observed (Fig 3K) and the number of Mf in lungs correlated with blood microfilaremia (Fig 3l). Histologically, Mf were quantified in various lung compartments (Fig 3M): Mf were observed mainly in lung blood circulation (veins, arteries and capillaries—Fig 3N). They were also observed in perivascular spaces (PVS) which comprise of the interstitial collagenous sheath surrounding larger veins and arteries in the lung (Fig 3O), and occasionally in alveoli.

Taken together, it seems likely that the increased numbers of Mf are due to: 1) an increased survival of adult parasites leading to female parasites laying eggs for a longer period of time; 2) better development of adult worms with more fertile females; and 3) enhanced production of Mf with more eggs laid.

### Damage of both bronchial epithelium and visceral pleura in microfilaremic mice is dependent on IL-4R and/or eosinophils

**Fig 4:**
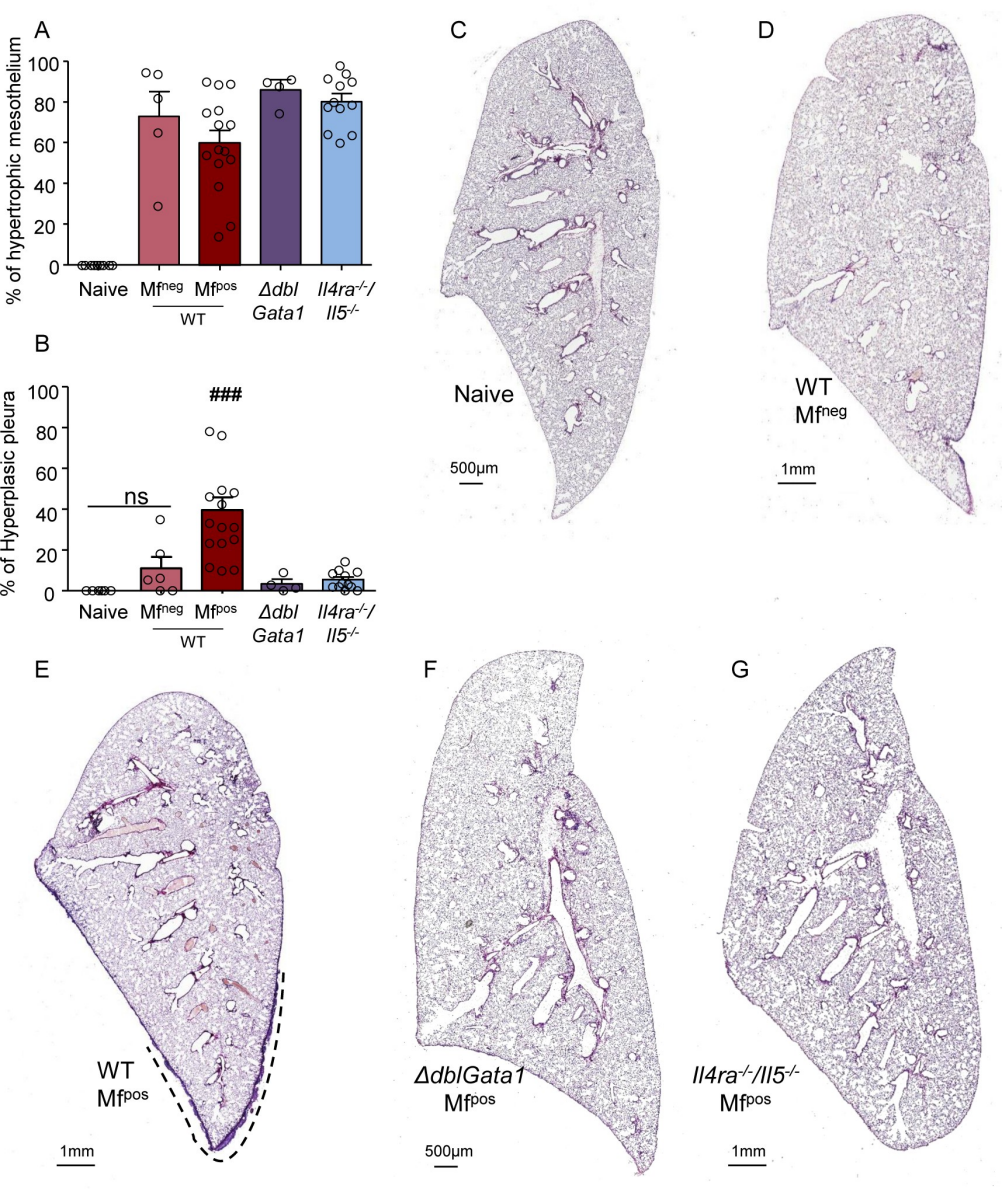
Absence of IL-4R and/or eosinophils protects from visceral pleura hyperplasia in microfilamic mice. *L*. *sigmodontis* infected wild type (WT), *ΔdblGata1* and *Il-4ra*^*-/-*^*/Il-5*^*-/-*^ BALB/c mice were euthanized at 70 days p.i. Hematoxylin and Sirius red staining of left lung sections was performed to analyze the visceral pleura. (**A**) Proportion of visceral pleura with hypertrophic mesothelium. (**B**) Proportion of hyperplasic visceral pleura; results are expressed as mean ± SEM (pool of 2–3 experiments): n = 10 naive, n = 7 WT Mf^neg^, n = 15 WT Mf^pos^, n = 4 ΔdblGATA- Mf^pos^, n = 12 *Il-4ra*^*-/-*^*/Il-5*^*-/-*^. Kruskal-Wallis followed by a Dunns multiple comparison test: **###**p<0.001 represent difference between naive and infested (C-G) Representative left lung whole section of (**C**) naive mice; (**D**) WT Mf^neg^ mice; (**E**) WT Mf^pos^ mice. The dashed line shows the hyperplasic area of the visceral pleura; (**F**) *ΔdblGata1* Mf^pos^ mice; (**G**) *Il-4ra*^*-/-*^*/Il-5*^*-/-*^ Mf^pos^ mice.

Histological analysis of the lungs was performed in the different groups of mice (Fig 4C–4G) to stratify the inflammatory state of the visceral pleura according to Mf status and genotype. All groups of infected mice presented a large portion of the visceral pleura (60 to 80%) covered with hypertrophic mesothelial cells (Fig 4A) but strong hyperplasia was only observed in WT Mf^pos^ mice (Fig 4B and 4E).

**Fig 5:**
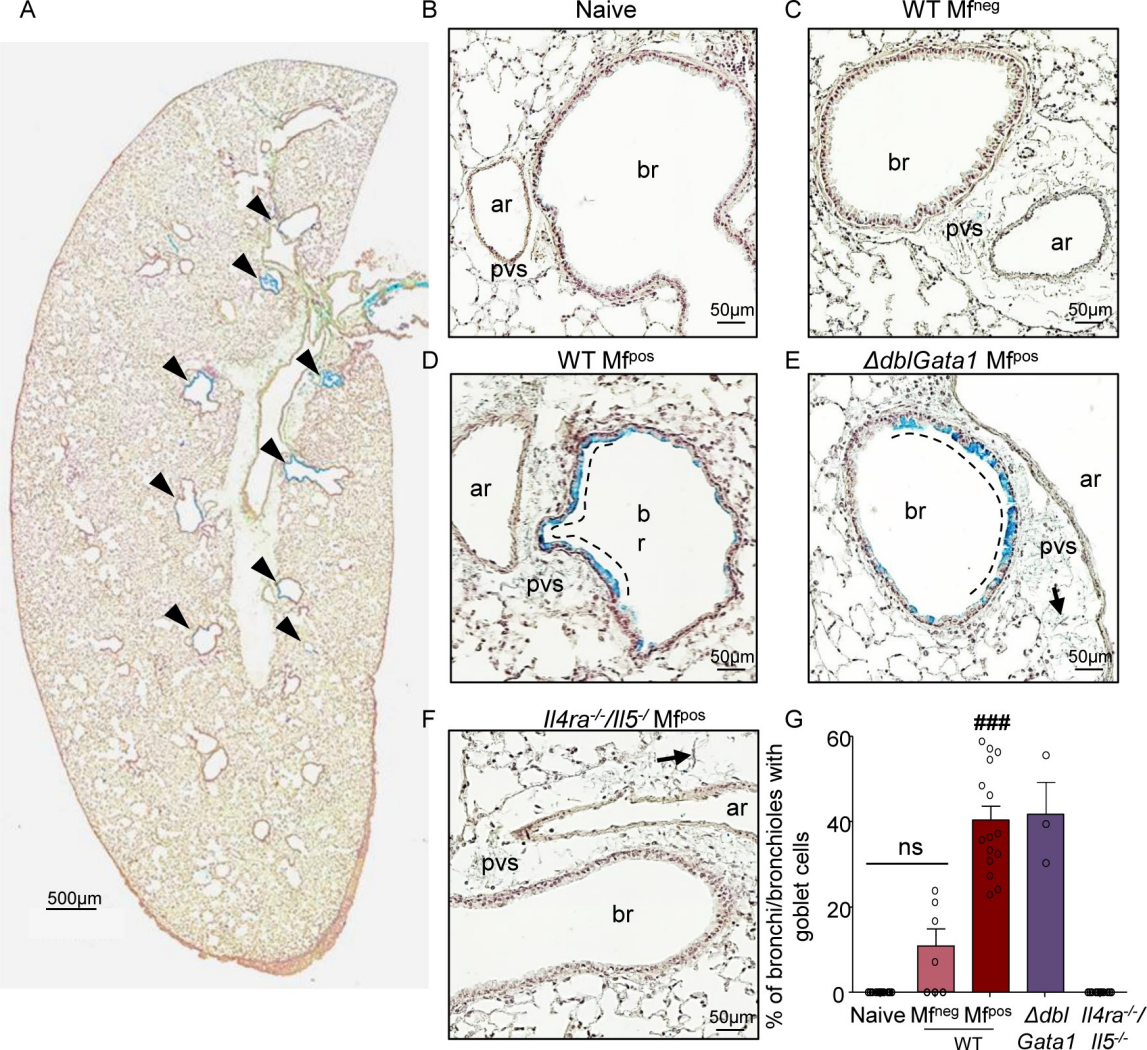
Absence of IL-4R impairs bronchial inflammation in microfilaremic mice. *L*. *sigmodontis* infected wild type (WT), *ΔdblGata1* and *Il-4ra*^*-/-*^*/Il-5*^*-/-*^ BALB/c mice were euthanized at 70 days p.i. Alcian Blue-PAS staining of left lung sections was performed to analyse the mucus secretion. (**A**) Representative left lung whole section of a WT Mf^pos^ mouse showing mucus-producing bronchial epithelium (arrow head) and a thickened posterior visceral pleura. Bronchial epithelium lacking mucus producing goblet cells in (**B**) naïve, (**C**) WT Mf^neg^ and (**F**) Mf^pos^ *Il-4ra*^*-/-*^*/Il-5*^*-/-*^ mice. Presence of mucus-producing goblets cells in bronchial epithelium of (**D**) WT Mf^pos^ and (**E**) *ΔdblGata1* Mf^pos^ mice with preferential localization of goblet cells close to perivascular space (pvs). Arrows show Mfs in pvs; br = bronchus; ar = artery (**G**) Percentage of bronchi containing mucus-producing goblet cells; results are expressed as mean ± SEM (pool of 1–3 experiments). n = 10 naive, n = 7 WT Mf^neg^, n = 15 Mf^pos^, n = 3 *ΔdblGata1* Mf^pos^, n = 12 *Il-4ra*^*-/-*^*/Il-5*^*-/-*^. For each mouse, 2–3 lung sections were analyzed. Kruskal-Wallis followed by a Dunns multiple comparison test: **###**p<0.001 represent difference between naive and infested.

Mucus production, as an indicator of lung inflammation, was assessed (Fig 5). Triggering of the IL-4R by IL-4, and particularly by IL-13, is the main cause of goblet cell metaplasia [25, 26]. No goblet cells were present in the bronchial epithelium of naive mice (Fig 5B and 5G) and only a few were observed in bronchial epithelium of WT Mf^neg^ mice (Fig 5C and 5G). However, goblet cells were substantially increased in the bronchial epithelium of WT and *ΔdblGata1* Mf^pos^ mice (Fig 5A, 5D, 5E and 5G) revealing mucus production in these two groups of mice. These cells were mainly located adjacent to PVS (Fig 5D and 5E) and absent in the bronchial epithelium of *Il-4ra*^*-/-*^*/Il-5*^*-/-*^ Mf^pos^ mice (similar to naive mice) (Fig 5F and 5G).

### Inflammation is increased in the pleural cavity and the bronchoalveolar space of infected or microfilaremic mice

The pleural cavity and bronchoalveolar space of the lungs are both known to be important in controlling the filarial burden by providing cells, cytokines, chemokines and other growth factors [13, 17, 18].

**Fig 6:**
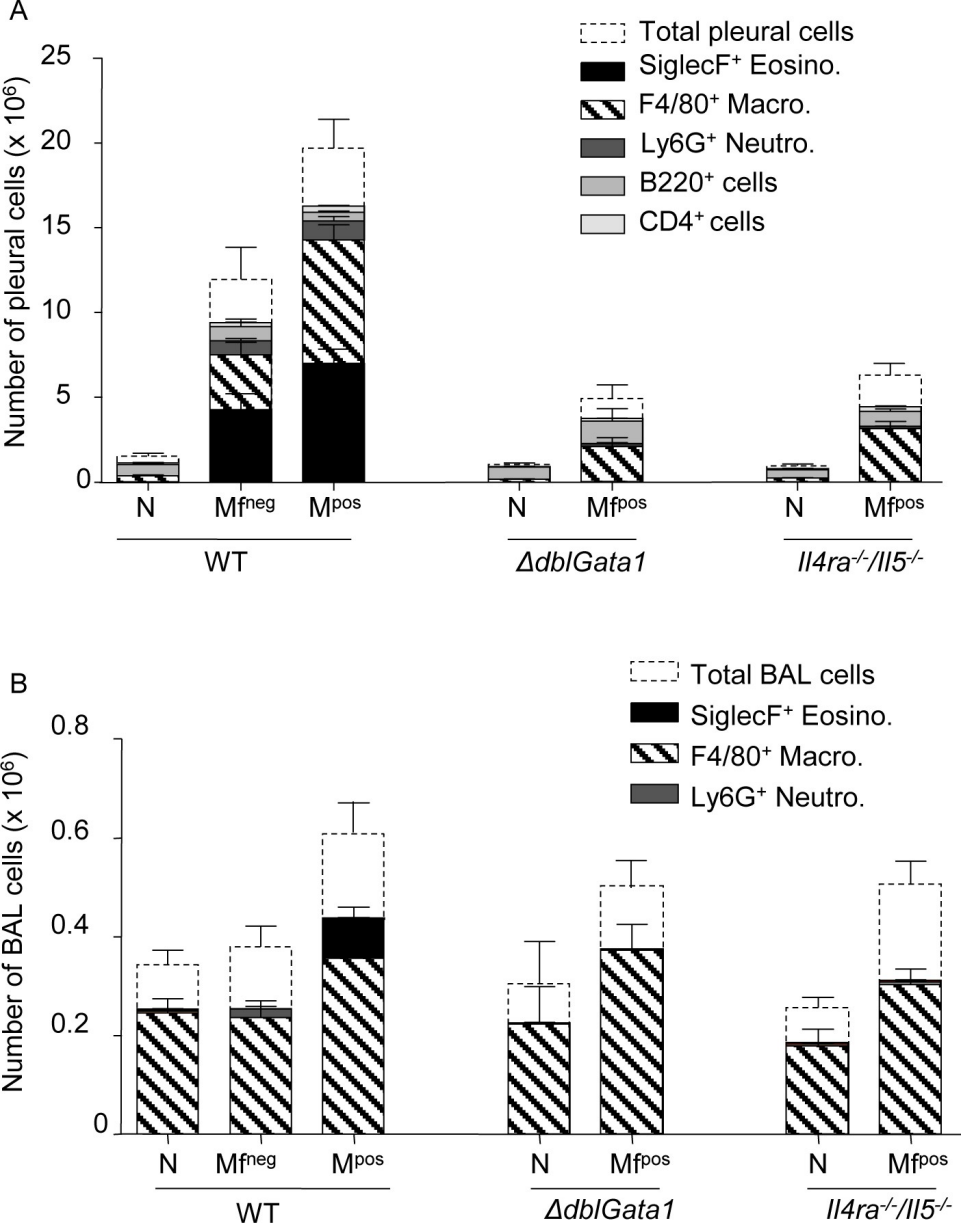
Cells increase in the pleural cavity of infected mice and in the bronchoalveolar space of microfilaremic mice. Pleural **(A)** and bronchoalveolar **(B)** cells were isolated from *L*. *sigmodontis* infected WT, *ΔdblGata1* and *Il-4ra*^*-/-*^*/Il-5*^*-/-*^ BALB/c mice at 70 days p.i. Cell phenotypes (SiglecF^+^ eosinophils, F4/80^+^ macrophages, Ly6G^+^ eosinophils, CD19^+^ B-cells and CD4^+^ T-cells) were analyzed by flow cytometry. Results are expressed as mean ± SEM (pool of 2–4 independent experiments for pleural cells; pool of 2–3 independent experiments for bronchoalveolar cells): n = 13–18 WT naive, n = 10–16 WT Mf^neg^, n = 21–28 WT Mf^pos^, n = 2 *ΔdblGata1* naive, n = 6 *ΔdblGata1* Mf^pos^, n = 6–15 *Il-4ra*^*-/-*^*/Il-5*^*-/-*^ naive, n = 17 *Il-4ra*^*-/-*^*/Il-5*^*-/-*^ Mf^pos^.

Filarial infection in WT mice induced a significant increase in cell numbers in the pleural cavity (PC) where the adult worms are located (Fig 6A, S2A–S2D Fig) with an increase in eosinophils, macrophages and neutrophils that was even more pronounced in Mf^pos^ mice. Mf dependent changes were also observed in the bronchoalveolar lavage (BAL). In contrast with the PC, increased bronchoalveolar macrophages were observed in all three groups of Mf^pos^ mice (Fig 6B, S2G Fig). Additionally, an increase in eosinophils in the bronchoalveolar space of WT Mf^pos^ mice only was also observed (Fig 6B, S2F Fig). Only a slight increase in the number of F4/80^+^ macrophages was observed in *ΔdblGata1* and *Il-4ra*^*-/-*^*/Il-5*^*-/-*^mice *versus* naïve mice. Similarly, many cytokines were also increased depending on the presence of adult worms or Mf (S3A–S3K Fig). The inflammatory cytokine IL-6 was increased in the PC of both infected WT groups as well as of Mf^pos^ *ΔdblGata1* mice but not in the PC of *Il-4ra*^*-/-*^*/Il-5*^*-/-*^ mice (S3A Fig). However IL-6 was not modified in the BAL of any groups of mice (S3G Fig). The monocyte / macrophage-chemotactic and pro-fibrotic chemokine CCL2 was increased in the PC and the BAL of all the infected mice independently of the presence of Mf (S3B Fig). The Th2 cytokine IL-4, necessary for alternative activation of macrophages, was increased in PC of all infected mice (S3C Fig). Increased IL-4 was found in the PC of *Il-4ra*^*-/-*^*/Il-5*^*-/-*^ mice (including naive *Il-4ra*^*-/-*^*/Il-5*^*-/-*^ mice; S3C Fig). However, no significant differences were observed in the levels of IL-4 in the BAL of any groups of mice (S3I Fig). Regulation of eosinophil maturation, recruitment, and survival is under the control of a small group of factors, including IL-5 and CCL11. Although IL-5 was not detected, CCL11 was increased in the three groups of Mf^pos^ mice, with lower levels in *Il-4ra*^*-/-*^*/Il-5*^*-/-*^ mice (S3D Fig). Again, IL-5 was not detected in BAL of any groups of mice but CCL11 was increased in the BAL of all infected groups (S3J Fig). CXCL9 was increased in all the PC of the three groups of Mf^pos^ mice but *Il-4ra*^*-/-*^*/Il-5*^*-/-*^ mice had higher levels (S3E Fig). In BAL, CXCL9 was increased only in Mf^pos^ mice (S3K Fig).

As this could be explained by IFN-ɣ driven CXCL9 production, we also checked IFN-ɣ levels in the pleural cavity and observed that they increased in all groups of Mf^pos^ mice (S3F Fig).

### Inflammation in the pleural cavity and bronchoalveolar space is associated with a specific activation of macrophages

**Fig 7:**
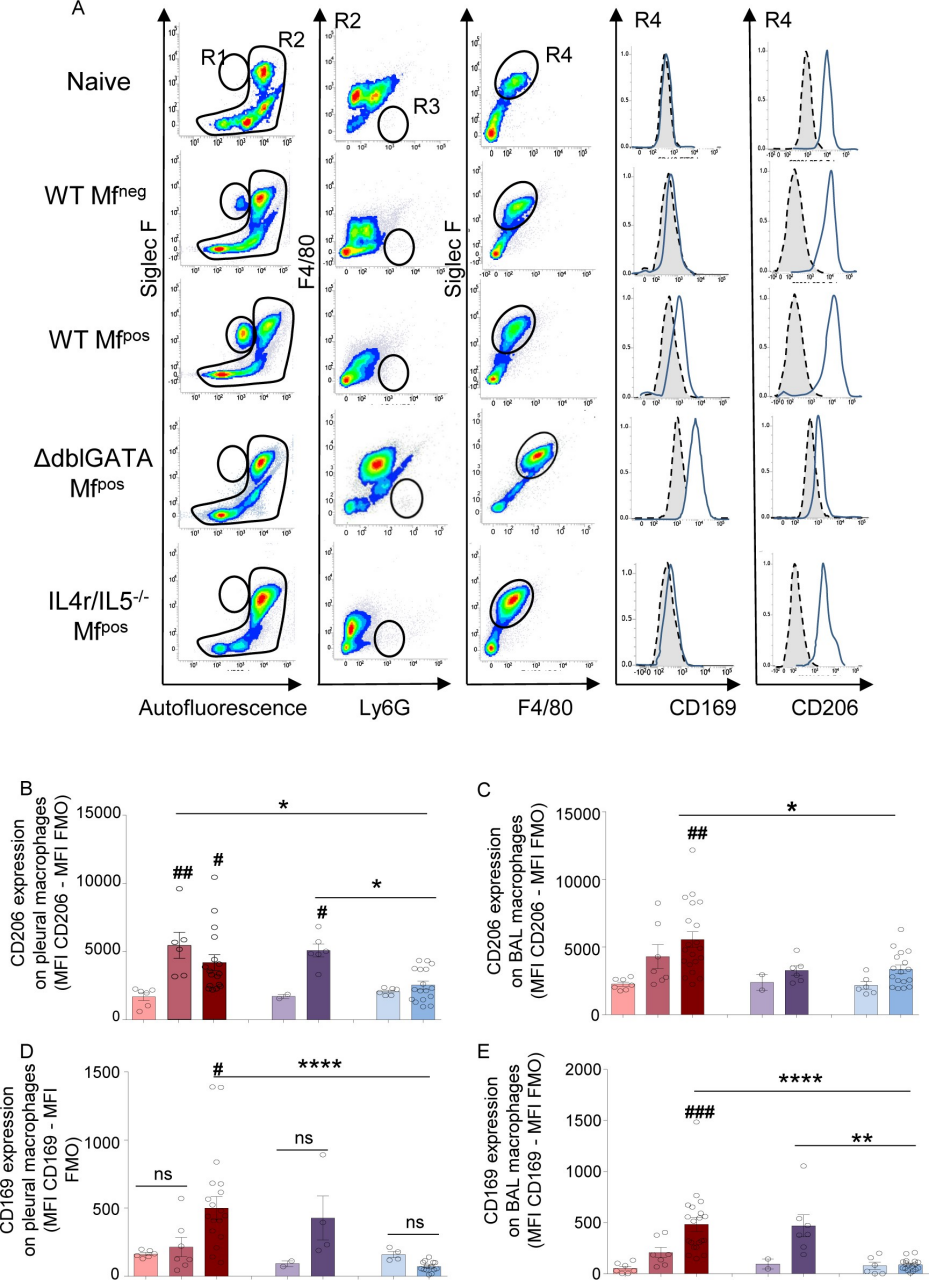
Activation of macrophages in infected mice is dependent on IL-4R and/or eosinophils. Pleural and bronchoalveolar cells were isolated from *L*. *sigmodontis* infected WT, *ΔdblGata1* and *Il-4ra*^*-/-*^*/Il-5*^*-/-*^ BALB/c mice at 70 days p.i. Macrophage activation (CD206 and CD169 expression) were analyzed by flow cytometry. (**A**) Representative plots and curves showing the gating strategy for bronchoalveolar cells stained with anti-SiglecF-PE, anti-F4/80-APC, anti-Ly6G-V450, anti-CD169-FITC and anti-CD206-PE-Cy7. Doublets and dead cells were excluded prior further analysis. R1 = SiglecF^+^ eosinophils. R2 = Non-eosinophil cells. R3 and R4 were defined from the R2 population: R3 = Ly6G^+^ neutrophils; R4 = F4/80^+^ macrophages; CD206 and CD169 expressions were measured on R4 population (F4/80^+^ macrophages). Gray area represents the FMO signal. **(B)** CD206 expression on pleural macrophages; (**C**) CD206 expression on bronchoalveolar macrophages; **(D)** CD169 expression on pleural macrophages; (**E**) CD169 expression on bronchoalveolar macrophages. Both CD206 and CD169 expressions were normalized by subtracting the FMO signal. Results are expressed as mean ± SEM of n = 6–7 WT naïve, n = 7 WT Mf^neg^, n = 19–20 WT Mf^pos^; n = 2 *ΔdblGata1* naïve; n = 5–7 *ΔdblGata1* Mf^pos^; n = 6 *Il-4ra*^*-/-*^*/Il-5*^*-/-*^ naive; n = 17 *Il-4ra*^*-/-*^*/Il-5*^*-/-*^ Mf^pos^. Differences between infected groups and respective naïve groups were analyzed by a One-way ANOVA (after checking the conditions of application of the test): **#**p<0.05, **##**p<0.01, **###**p<0.001. Differences between Mf^pos^ groups *p<0.05, **p<0.01, ****p<0.0001 groups were also analyzed by a One-way ANOVA.

Specific activation of macrophages was investigated in both compartments, pleural cavity (PC) and bronchoalveolar space (Fig 7A–7E). Expression of the mannose receptor, CD206, a marker that has been associated with a pro-repair phenotype [27] was increased on pleural macrophages from infected WT Mf^neg^, WT Mf^pos^ and *ΔdblGata1* Mf^pos^ mice, but not on those from *Il-4ra*^*-/-*^*/Il-5*^*-/-*^ Mf^pos^ mice (Fig 7B), confirming an IL-4R-dependent phenotype in these macrophages [28]. CD206 was only increased on airway macrophages from WT Mf^pos^ mice (Fig 7C). Interestingly, CD169, a marker related to a subpopulation of macrophages described under inflammatory conditions [29] was uniquely increased on pleural macrophages from infected WT Mf^pos^, but not Mf^neg^ mice expressing the IL-4R (Fig 7D). As in PC, CD169 expression was increased on bronchoalveolar macrophages from Mf^pos^ mice expressing the IL-4R, i.e. WT Mf^pos^ and *ΔdblGata1* Mf^pos^ mice (Fig 7E).

### IL-4R-dependent increase in specifically localized tissue-resident CD169^+^ macrophages in microfilaremic mice

**Fig 8:**
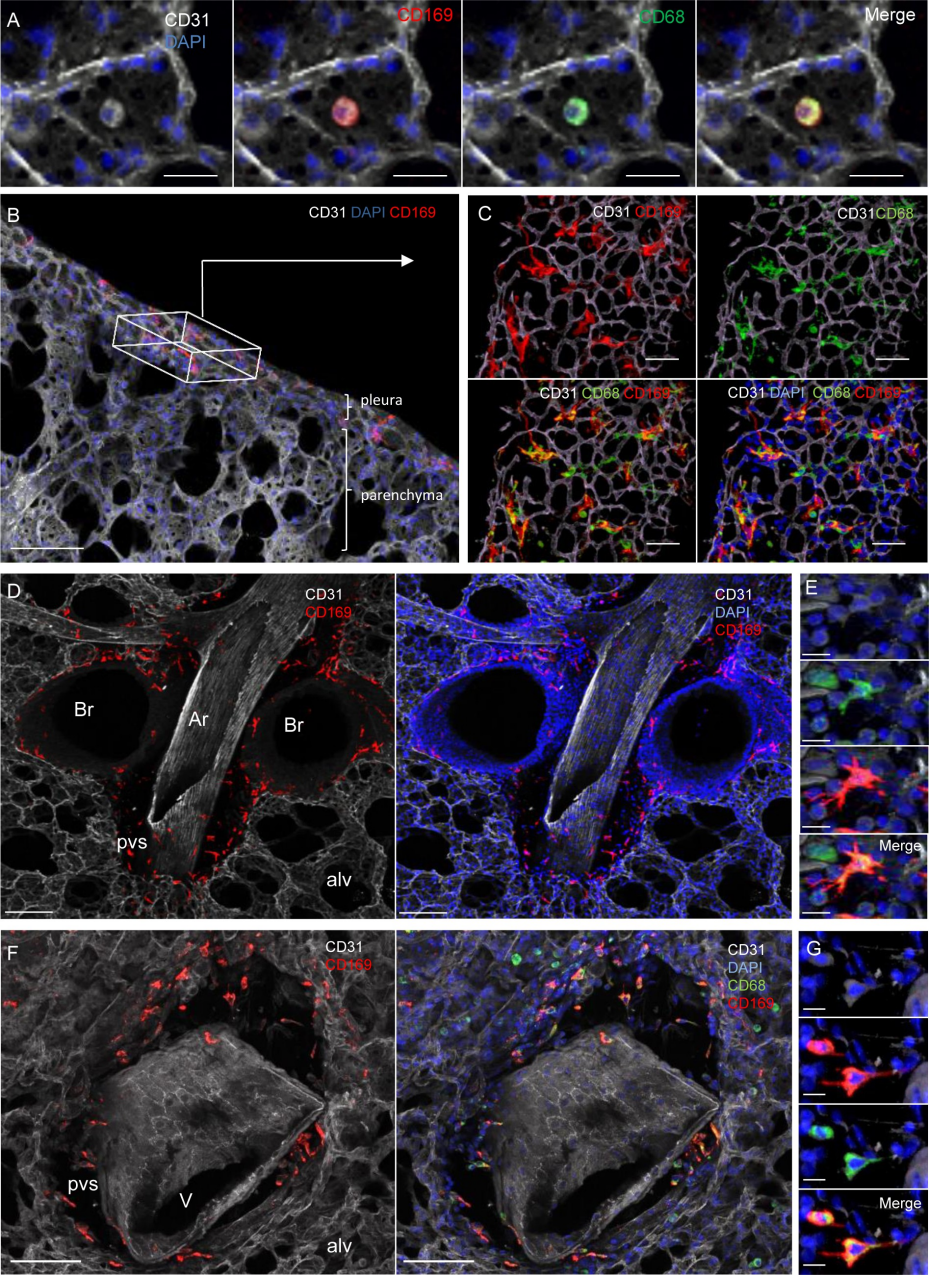
CD169 expression on resident macrophage populations in specific lung locations. Lungs were recovered from naive and *L*. *sigmodontis* infected WT, *ΔdblGata1* and *Il-4ra*^*-/-*^*/Il-5*^*-/-*^ BALB/c mice at 70 days p.i.. Precision cut lung slices (PCLS) were prepared for confocal microscopy analysis. Representative maximum intensity projection from confocal z-stacks (z = 50μm) of PCLS stained with DAPI (blue) and fluorochrome-conjugated anti-CD31 (white), -CD68 (green) and -CD169 (red) antibodies; (**A**) Alveolus (surrounded by CD31^+^ capillaries) containing a CD68^+^CD169^+^ alveolar macrophage; Left: DAPI and CD31 channels; Middles: DAPI and CD31 with CD169 and CD68 single channels respectively; Right: Merging of the different channels showing the coexpression of CD169 and CD68 in alveolar macrophages; Scale bar = 20μm; (**B**) 50μm stack view of lung periphery showing CD169^+^ cells (red) in the visceral pleura. The box indicates pleura orientation. Scale bar = 100μm; (**C**) Top view of the visceral pleura showing CD169 and CD68 expression macrophages close to CD31^+^ capillaries. Top: CD169 and CD68 single channels with CD31. Bottom: Merging of the different channels with DAPI. Scale bar = 20μm; (**D**) CD31^+^ artery surrounded by a perivascular space (pvs) and adjacent to bronchi. Pvs and peribronchial space contain CD169^+^ cells. Scale bar = 100μm. (**E**) Higher magnification of a macrophage in a pvs around an artery showing expression of CD68 (green) and CD169 (red). Scale bar = 10μm; Top: DAPI and CD31 channels; Middle: CD169 and CD68 single channels with DAPI and CD31; Bottom: Merging of the different channels showing the coexpression of CD68 and CD169 in macrophages in artery pvs (**F**) CD31^+^ vein (identified by round nuclei and absence of adjacent bronchus) surrounded by a perivascular space (pvs) containing CD68^+^CD169^+^ cells. Scale bar = 100μm (**G**) Higher magnification of a macrophage in a pvs around a vein showing expression of CD68 (green) and CD169 (red). Scale bar = 10μm. Top: DAPI and CD31 channels; Middle: CD68 and CD169 single channels with DAPI and CD31; Bottom: Merging of the different channels showing the coexpression of CD169 and CD68 in macrophages in vein pvs. br: bronchus; alv: alveolus; Ar: artery; V: vein; pvs: perivascular space.

Considering the data above, our next step was to localize CD169-expressing lung bronchoalveolar and interstitial macrophages by imaging agarose inflated precision-cut lung slices (PCLS). CD68^+^CD169^intermediate^ bronchoalveolar (airway) macrophages were observed in all groups of mice (Fig 8A). Strikingly, based on specific localization, four additional tissue-resident groups of CD68^+^CD169^+^ interstitial macrophages (IM) with particularly bright CD169 staining were identified in the lungs of naive and infected mice (Fig 8). These cells localized: 1) in the periphery of the lung along the visceral pleura (Fig 8B and 8C); 2) in the interstitium surrounding bronchi just below bronchial epithelium (Fig 8D); 3) in the PVS surrounding arteries (Fig 8D and 8E); and 4) surrounding veins (Fig 8F and 8G).

**Fig 9:**
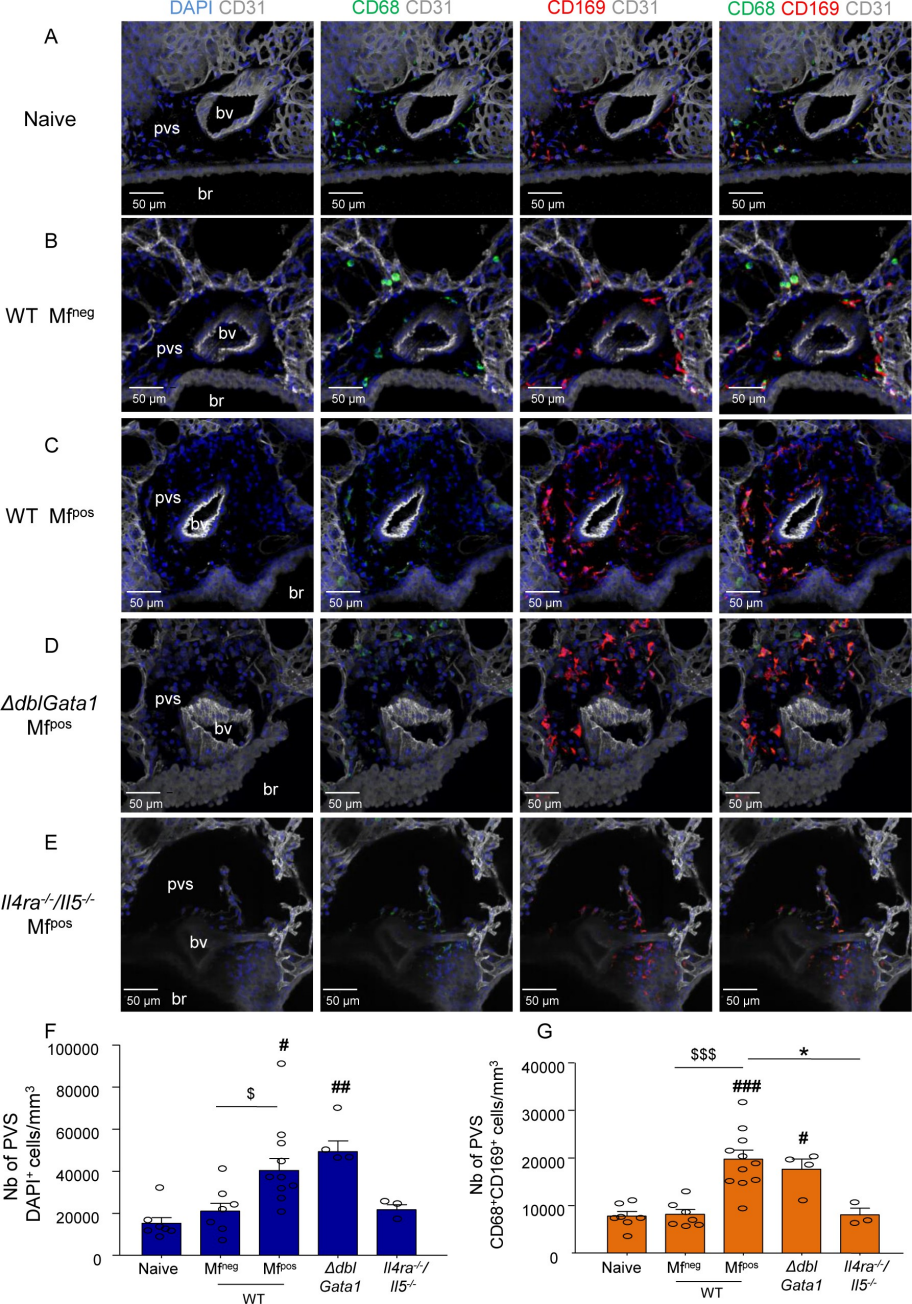
IL-4R/IL-5 dependent increase of lung-resident CD169^+^ macrophages in microfilaremic mice. Lungs were recovered from naive and *L*. *sigmodontis* infected WT, *ΔdblGata1* and *Il-4ra*^*-/-*^*/Il-5*^*-/-*^ BALB/c mice at 70 days p.i.. Precision cut lung slices (PCLS) were prepared for confocal microscopy analysis. (**A-E**) Representative maximum intensity projection from confocal z-stacks (z = 50μm) of PCLS stained with DAPI (blue) and fluorochrome-conjugated anti-CD31 (white), -CD68 (green) and -CD169 (red) antibodies. Analysis of perivascular space (PVS) cellular content in the different groups of mice: Left column: DAPI and CD31 channels; Middle columns: DAPI and CD31 with CD169 and CD68 single channels respectively; Right column: Merging of the different channels showing CD68^+^CD169^+^ macrophages in PVS; (**A**) PVS of a naive uninfected mouse (**B**) PVS of a WT Mf^neg^ mouse; (**C**) PVS of a WT Mf^pos^ mouse; (**D**) PVS of a *ΔdblGata1* Mf^pos^ mouse; (**E**) PVS of a *Il4ra*^*-/-*^*/Il5*^*-/-*^ Mf^pos^ mouse; (**F-G**) Quantification of PVS cellular content. PVS cells were counted and PVS volume was measured over the 50μm z-stack (using IMARIS software) to evaluate cell concentrations. (**F**) total number of cells (DAPI^+^) per mm^3^ of PVS and (**G**) number of CD68^+^CD169^+^ macrophage per mm^3^ of PVS. Results are expressed as mean ± SEM (pool of 1–3 experiments) of n = 7 naive, n = 7 WT Mf^neg^, n = 11 Mf^pos^, n = 11, n = 4 *ΔdblGata1* Mf^pos^, n = 4 *Il-4ra*^*-/-*^*/Il-5*^*-/-*^ Mf^pos^. For each mouse, 2–3 pvs were analyzed. Kruskal-Wallis followed by a Dunns multiple comparison test: **#**p<0.05, **##**p<0.01, **###**p<0.001 represent differences between infested groups and the naïve group; $p<0.05, $ $ $p<0.001 represent differences between Mf^neg^ and Mf^pos^ mice *p<0.05 represent difference between Mf^pos^ groups. bv = blood vessel; br = bronchus.

Subsequently PCLS from WT and mutant mice were imaged to further analyze the PVS (Fig 9A–9E) and quantify its cellular content (Fig 9F and 9G). The PVS of naive mice contained few cells (DAPI^+^) 50% of which were CD68^+^CD169^+^ IM (Fig 9A). WT Mf^neg^ mice had a similar cell content (Fig 9B). However, PVS cell content of WT Mf^pos^ and *ΔdblGata1* Mf^pos^ was markedly 2–3 times higher than that of naive or WT Mf^neg^ mice. CD68^+^CD169^+^ IM were 30–40% of the total cell content in WT Mf^pos^ and *ΔdblGata1* Mf^pos^ (Fig 9C and 9D). Interestingly, these changes were abrogated in the PVS of *Il-4ra*^*-/-*^*/Il-5*^*-/-*^ Mf^pos^ mice (Fig 9E).

## Discussion

Pulmonary pathology in filariasis is underestimated compared with the main manifestations of human filariases such as lymphoedemas or ocular pathologies. For example, both human *Mansonella perstans* and rodent *Litomosoides sigmodontis* can be found in the pleural cavity (PC) and they are both considered asymptomatic even if local PC inflammation has been described [5, 17]. However, analysis of lung tissue has barely been performed. Here, we document a pulmonary pathology in microfilaremic (Mf^pos^) *L*. *sigmodontis*-infected rodents. Common features with TPE due to the Mf of *B*. *malayi* and *W*. *bancrofti* were observed: the presence of bronchoalveolar eosinophils and increases lung macrophage numbers, the production of mucus and the occurrence of pulmonary fibrosis [4, 10]. Moreover, the development of lung pathology, the parasite survival, growth and fertility, as well as the microfilaremic status are conditioned by a Th2 environment in the murine host.

Many patients infected by LF or mansonellosis are amicrofilaremic (Mf^neg^) [2, 5, 14] and similarly 40% of *L*. *sigmodontis* infected BALB/c mice are Mf^neg^ mice. A few immune components have been identified as key players in controlling the microfilarial burden of mice (S2 Table). Filarial fertility is clearly controlled by Th2 responses as infections of *ΔdblGata1* (eosinophil deficient) and *Il-4ra*^*-/-*^*/Il-5*^*-/-*^ (wider Th2 deficiency including defect in macrophage alternative activation) BALB/c mice result in 80 to 100% Mf^pos^ mice [23] with 10 to 35 times higher microfilaremia. This is associated with an increase in the survival of filariae but also a better growth of the parasites and a more successful oogenesis.

Both eosinophils and macrophages are known to be essential for the elimination of adult worms and Mf [17, 30, 31]. The decrease/absence of these cells in both mutant mice could thus explain the increased parasite survival. However even if the number of these cells is important their activation is also decisive. *L*. *sigmodontis* infection is known to induce an alternative activation macrophage-type phenotype (AAM) in the PC of mice [32, 33] under the control of IL-4R [28]. Such an activation is independent of the microfilaremic status as an increase of CD206 expression was observed in all competent mice. CD169 has also been associated with an activation of a subset of macrophages under inflammatory conditions [34]. Together, increased CD206 and CD169 on human bronchoalveolar macrophages have been documented in a fibrotic setting [35]. Here Mf^pos^ mice show an increased CD169 expression suggesting a role for CD169^+^ macrophages in the Mf-driven pathology. IFN-γ is known to induce CD169 expression on monocytes [29] and its increase in Mf^pos^ mice could be responsible for the activation of CD206^+^ AAM. CD169 (known as Siglec1/Sialoadhesin/MOMA-1) is a lectin receptor mediating the binding to neutrophils, innate lymphoid cells and dendritic cells or pathogens through sialylated glycoproteins and glycolipids [36]. *L*. *sigmodontis* Mfs are surrounded by a sheath containing such acids [37], so it is possible that CD169 would help macrophages to adhere to Mfs to allow their phagocytosis.

CD169 expression in lungs was previously thought to be specific for bronchoalveolar macrophages [38] even if some former reports noticed the presence of CD169^+^ cells in interstitial spaces [39, 40]. We confirm that mouse lungs contain several groups of CD169-expressing tissue-resident macrophages with specific localizations. CD169^+^ tissue-resident macrophages were located at every interface area of the lungs, *i*.*e*. in alveoli (alveolar macrophages) and in the visceral pleura, around bronchi and blood vessels (interstitial macrophages; IM) and strikingly in these spaces, all the macrophages were CD169^+^. The increase of IM in Mf^pos^ mice could be due to a local proliferation of the macrophages [39]. Based on their prominent morphology and localization these cells are almost certainly the same tissue resident lung macrophages identified as likely to be yolk sac-derived cells with self-renewal properties in Runx1 lineage tracing experiments [41, 42].

The increase of immune cells in the perivascular space (PVS) potentially informs us on the function of this anatomical structure. It is a connective tissue composed of extracellular matrix (ECM) which has a clear structural function in the maintenance of lung architecture but also in the migration of leukocytes [43]. Indeed ECM collagen fibers present in PVS may provide an attachment point for leukocyte motility around these pulmonary arteries or veins [44]. Accumulation of leukocytes has been observed in the PVS in models of lung infections, fibrosis, and allergic reactions [45–47]. It was also noticed in biopsies of patients affected by TPE [48]. Among these cells, CD169^+^ perivascular cells were increased during infection by another worm *Schistosoma mansoni* [49]. The function of such accumulation remains elusive. Because of their localization near blood vessels and airways, the cells present in PVS may have a role in the management of exogenous molecules and microorganisms. In the kidney, CD169^+^ perivascular macrophages play an important role in controlling inflammation by limiting neutrophil influx into the tissue [50]. CD169^+^ tissue-resident macrophages can stimulate innate lymphoid cell [51] but also initiate CD8^+^ T cell responses by binding of CD169 to dendritic cells [52]. It is unknown how Mfs escape the blood circulation, but this extravascular location seems accidental as Mfs are no longer available for transmission to the vector. PVS cells (CD169^+^ IM and/or other cells like CD4 T-cells and innate lymphoid cells) could be responsible for producing an IL-13/IL-4 rich microenvironment in response to Mfs, leading to goblet cell metaplasia and proliferation/recruitment of PVS CD169^+^ IM.

In summary, these results suggest an unexpected role for CD169^+^ macrophages in response to Mf. They also support the use of chronic *L*. *sigmodontis* infection in Mf^pos^ mice as a model of TPE, challenges the classification of *L*. *sigmodontis* infection as asymptomatic and potentially informs us on lung pathology in *M*. *perstans* infection in humans.

## Supporting information

S1 Fig Life cycle of *Litomosoides sigmodontis*. Infective larvae (L3) are inoculated in the skin of the rodent host during a blood meal of the mite vector. L3 larvae migrate through the lymphatic system, then the pulmonary blood circulation to reach the pleural cavity within up to 8 days. At this level they will moult in L4 around 9–10 days post-infection (p.i.) and then in adult 30 days p.i.. Male and female parasites reproduce and release L1 larvae (microfilariae, Mf) in the pleural cavity approximately 55–60 days after infection. Mf reach the blood and are ingested by the mite vector during a blood meal. They moult in L2 stage in 5 to 7 days, then in L3 towards the 12^th^ day.
(TIF)

S2 Fig Phenotyping of pleural and bronchoalveolar cells. Pleural and bronchoalveolar cells were isolated from naive and *L*. *sigmodontis* infected WT, *ΔdblGata1* and *Il-4ra*^*-/-*^*/Il-5*^*-/-*^ BALB/c mice at 70 days p.i. Cells were analyzed by flow cytometry (FACSVerse flow cytometer) using fluorochrome-conjugated antibodies. **(A)** Total number of cells in pleural cavity. Absolute number of **(B)** SiglecF^+^ eosinophils **(C)** F4/80^+^ macrophages and (**D**) Ly6G^+^ neutrophils in the pleural cavity. **(E)** Total number of cell in the bronchoalveolar space. Absolute number of **(F)** SiglecF^+^ eosinophils, **(G)** F4/80^+^SiglecF^+^ macrophages and **(H)** Ly6G^+^ neutrophils in bronchoalveolar space. Results are expressed as mean ± SEM (pool of 2–4 independent experiments for pleural cells; pool of 2–3 independent experiments for bronchoalveolar cells): n = 13–18 WT naive, n = 10–16 WT Mf^neg^, n = 21–28 WT Mf^pos^, n = 2 *ΔdblGata1* naive, n = 6 *ΔdblGata1* Mf^pos^, n = 6–15 *Il-4ra*^*-/-*^*/Il-5*^*-/-*^ naive, n = 17 *Il-4ra*^*-/-*^*/Il-5*^*-/-*^ Mf^pos^. Kruskal-Wallis followed by a Dunn’s multiple comparison test: **#**p<0.05, **##**p<0.01, **###**p<0.001 represent differences between infested groups and respective naive groups; $p<0.05, $ $p<0.01 represent differences between Mf^neg^ and Mf^pos^ mice. *p<0.05, **p<0.01, ***p<0.001 represent differences between Mf^pos^ groups.
(TIF)

S3 Fig Cytokine analysis in the pleural cavity and the bronchoalveolar space. Pleural (Left) and Bronchoalveolar (right) fluids were isolated from *L*. *sigmodontis* infected WT, *ΔdblGata1* and *Il-4ra*^*-/-*^*/Il-5*^*-/-*^ BALB/c mice at 70 days p.i. (**A and G**) IL-6, (**B and H**) CCL2, (**C and I**) IL-4, (**D and J**) CCL11, (**E and K**) CXCL9 and (**F**) IFN-ɣ concentration were determined by ELISA. Results are expressed as mean ± SEM of 6 mice per group (n = 2 for naive *ΔdblGata1)*. Kruskal-Wallis followed by a Dunns multiple comparison test: **#**p<0.05, **##**p<0.01, **###**p<0.001 represent differences between infested groups and respective naive groups; $p<0.05 represent differences between Mf^neg^ and Mf^pos^ mice; *p<0.05 represent differences between Mf^pos^ groups.
(TIF)

S1 Table Pathway analysis of gene-related functions in infected lungs. Lungs were recovered from *L*. *sigmodontis* infected BALB/c mice at 70 days p.i. and processed for gene expression profiling of cytokines/chemokines. Pathway analysis results of gene-related functions in infected lungs (using IPA). The 1^st^ column indicates the high-level functional categories predicted to be activated; the 2^nd^ column precises the disease or function predicted to be activated; the 3^rd^ column shows the p-value calculated by IPA; the 4^th^ column gives the Activation z-score calculated by the IPA (activation if z-score ≥2); the 5^th^ column displays the molecules from the array involved in the diseases or functions; the 6^th^ one sums the molecules from the array involved in the disease or function.
(XLSX)

S2 Table Parasitological outcomes in different immunomodulated mice after *Litomosoides sigmodontis* infection. The 1^st^ column indicates the mouse strain; the 2^nd^ column precises the filarial developments according to the mouse strain; the 3^rd^ column indicates the molecular/cell target of the immunomodulation; the 4^th^ column gives the immunomodulatory tool (knock-out or transgenic mice, treatments with antibodies or drugs); the 5^th^ column indicates the main functional cell target; the 6^th^ one summarize the effect of the immunomodulation on the parasitological outcomes and the 7^th^ column is for the references. Mac: macrophages; Eos: eosinophils; Neu: neutrophils; rIL-5: recombinant IL-5; ↑: increase; ↓: decrease.
(DOCX)
