# Supplementary figures and images for: IL-4 receptor dependent expansion of lung CD169+ macrophages in microfilaria-driven inflammation
Source: PLoS Negl Trop Dis. 2019 Aug 30;13(8):e0007691. doi: 10.1371/journal.pntd.0007691 (PMC6742411; doi:10.1371/journal.pntd.0007691)

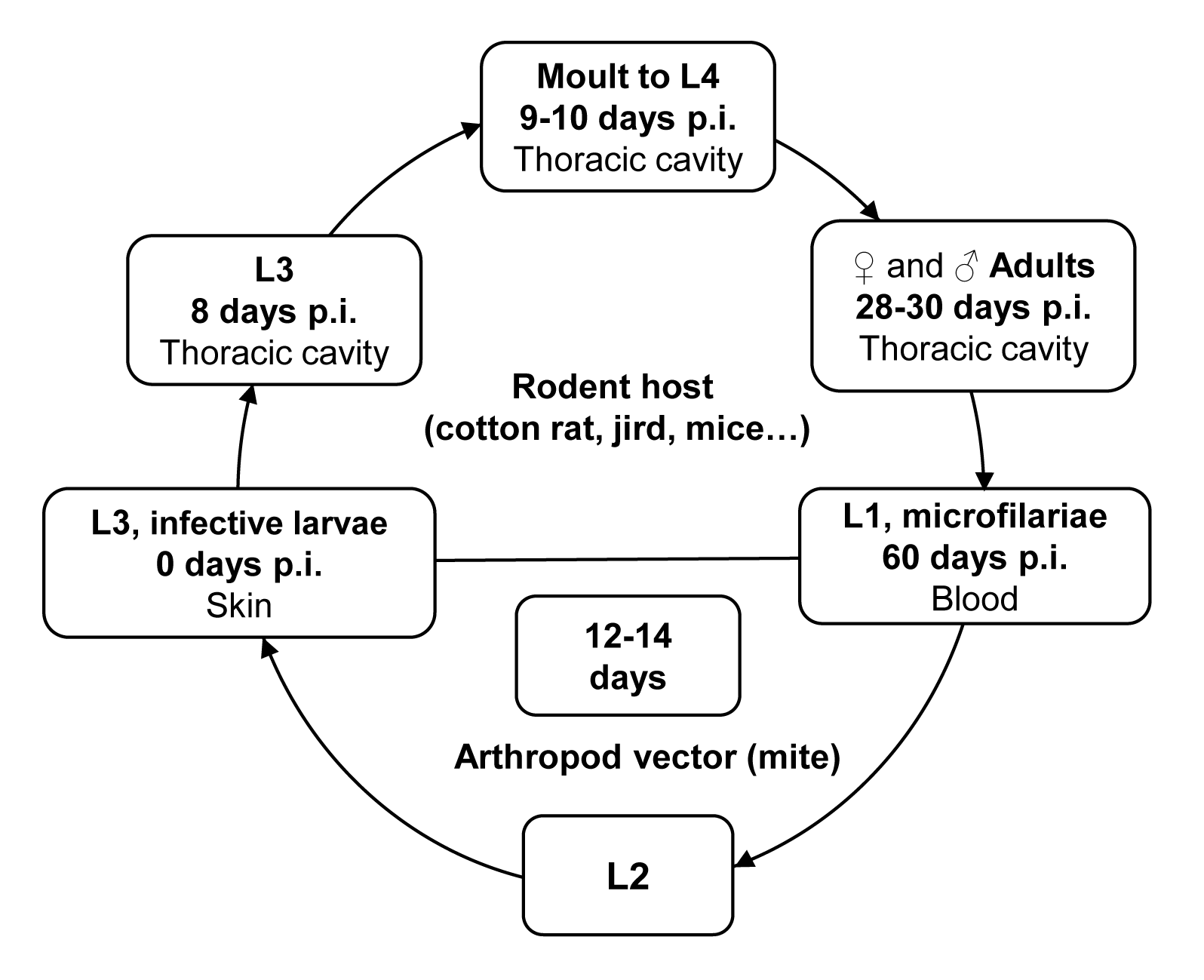

Supplement: S1 Fig — Infective larvae (L3) are inoculated in the skin of the rodent host during a blood meal of the mite vector. L3 larvae migrate through the lymphatic system, then the pulmonary blood circulation to reach the pleural cavity within up to 8 days. At this level they will moult in L4 around 9–10 days post-infection (p.i.) and then in adult 30 days p.i.. Male and female parasites reproduce and release L1 larvae (microfilariae, Mf) in the pleural cavity approximately 55–60 days after infection. Mf reach the blood and are ingested by the mite vector during a blood meal. They moult in L2 stage in 5 to 7 days, then in L3 towards the 12th day. (TIF) [file pntd.0007691.s001.tif]

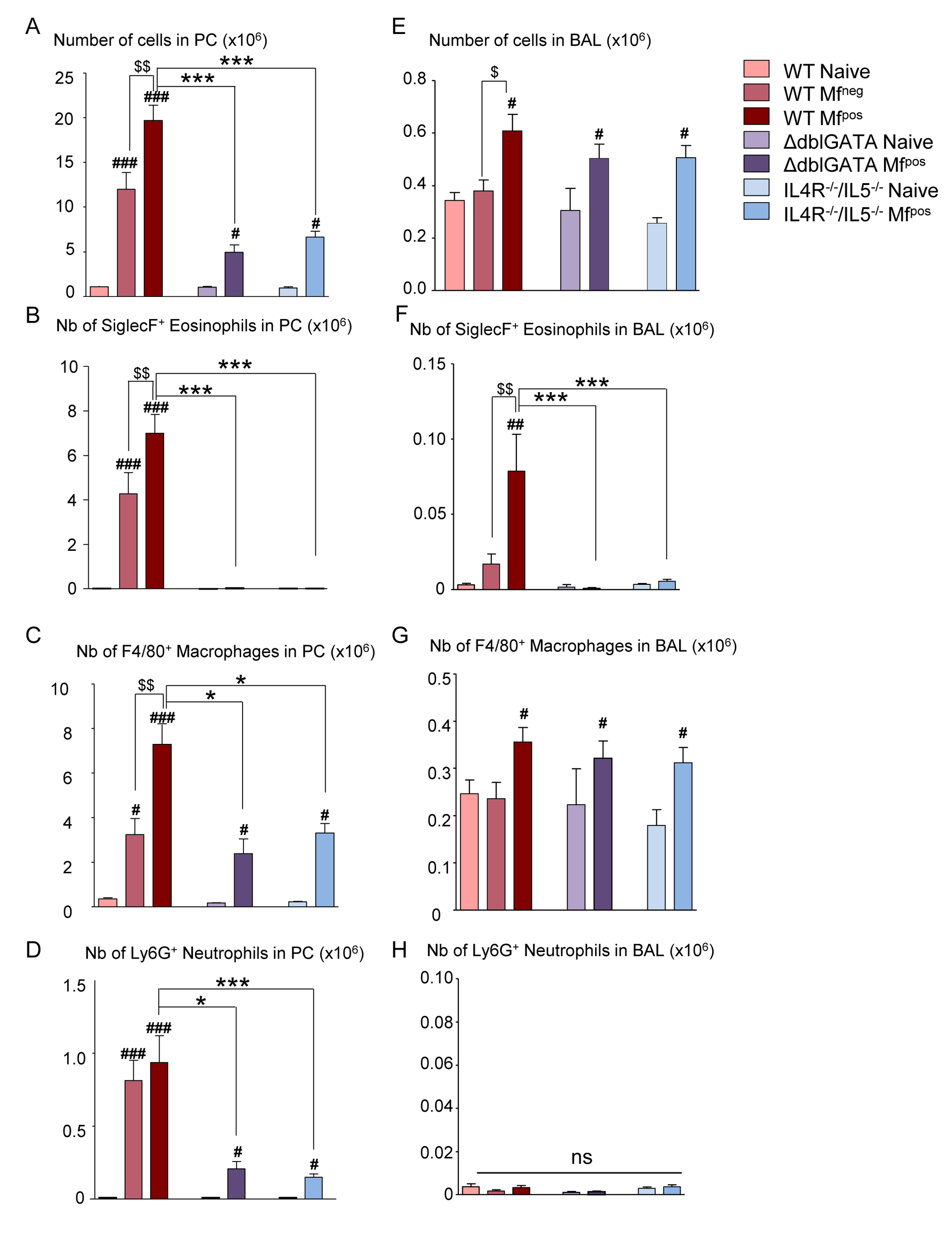

Supplement: S2 Fig — Pleural and bronchoalveolar cells were isolated from naive and L. sigmodontis infected WT, ΔdblGata1 and Il-4ra-/-/Il-5-/- BALB/c mice at 70 days p.i. Cells were analyzed by flow cytometry (FACSVerse flow cytometer) using fluorochrome-conjugated antibodies. (A) Total number of cells in pleural cavity. Absolute number of (B) SiglecF+ eosinophils (C) F4/80+ macrophages and (D) Ly6G+ neutrophils in the pleural cavity. (E) Total number of cell in the bronchoalveolar space. Absolute number of (F) SiglecF+ eosinophils, (G) F4/80+SiglecF+ macrophages and (H) Ly6G+ neutrophils in bronchoalveolar space. Results are expressed as mean ± SEM (pool of 2–4 independent experiments for pleural cells; pool of 2–3 independent experiments for bronchoalveolar cells): n = 13–18 WT naive, n = 10–16 WT Mfneg, n = 21–28 WT Mfpos, n = 2 ΔdblGata1 naive, n = 6 ΔdblGata1 Mfpos, n = 6–15 Il-4ra-/-/Il-5-/- naive, n = 17 Il-4ra-/-/Il-5-/- Mfpos. Kruskal-Wallis followed by a Dunn’s multiple comparison test: #p<0.05, ##p<0.01, ###p<0.001 represent differences between infested groups and respective naive groups; $p<0.05, $ $p<0.01 represent differences between Mfneg and Mfpos mice. *p<0.05, **p<0.01, ***p<0.001 represent differences between Mfpos groups. (TIF) [file pntd.0007691.s002.tif]

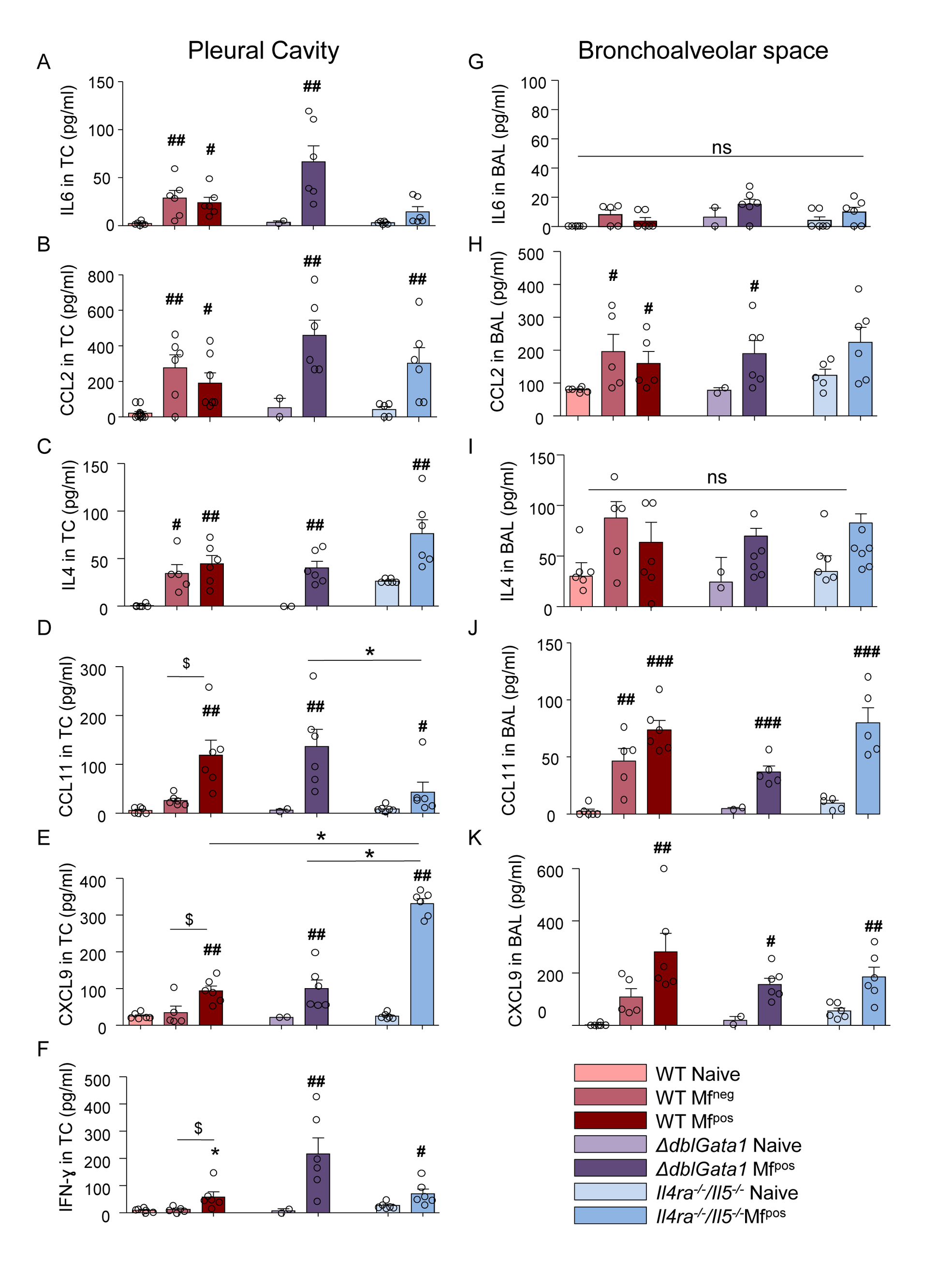

Supplement: S3 Fig — Pleural (Left) and Bronchoalveolar (right) fluids were isolated from L. sigmodontis infected WT, ΔdblGata1 and Il-4ra-/-/Il-5-/- BALB/c mice at 70 days p.i. (A and G) IL-6, (B and H) CCL2, (C and I) IL-4, (D and J) CCL11, (E and K) CXCL9 and (F) IFN-ɣ concentration were determined by ELISA. Results are expressed as mean ± SEM of 6 mice per group (n = 2 for naive ΔdblGata1). Kruskal-Wallis followed by a Dunns multiple comparison test: #p<0.05, ##p<0.01, ###p<0.001 represent differences between infested groups and respective naive groups; $p<0.05 represent differences between Mfneg and Mfpos mice; *p<0.05 represent differences between Mfpos groups. (TIF) [file pntd.0007691.s003.tif]
