# Supplementary material for: IL-4 receptor dependent expansion of lung CD169+ macrophages in microfilaria-driven inflammation
Source: PLoS Negl Trop Dis. 2019 Aug 30;13(8):e0007691. doi: 10.1371/journal.pntd.0007691 (PMC6742411; doi:10.1371/journal.pntd.0007691)
Supplement: S2 Table — The 1st column indicates the mouse strain; the 2nd column precises the filarial developments according to the mouse strain; the 3rd column indicates the molecular/cell target of the immunomodulation; the 4th column gives the immunomodulatory tool (knock-out or transgenic mice, treatments with antibodies or drugs); the 5th column indicates the main functional cell target; the 6th one summarize the effect of the immunomodulation on the parasitological outcomes and the 7th column is for the references. Mac: macrophages; Eos: eosinophils; Neu: neutrophils; rIL-5: recombinant IL-5; ↑: increase; ↓: decrease. (DOCX) [file pntd.0007691.s005.docx]

| Mouse strain | Filarial development | Target | Tool | Main functional cell target | Effect on patency | ref |
| --- | --- | --- | --- | --- | --- | --- |
| BALB/c | Sensible  (develop a patent phase) | IL4 | IL4^-/-^  mice | Mac | ­↑ microfilaremia | (1, 2) |
|  |  | IL4/IL13 | IL4R^-/-^  mice | Mac | ­↑ microfilaremia | (2) |
|  |  | IL5 | IL5^-/-^  mice | Eos | ­↑ Worm burden  ↑­ survival of adults ­↑ microfilaremia | (2,3) |
|  |  | IL4/IL13/IL5 | IL4R^-/-^ /IL5^-/-^  mice | Mac/Eos | ­↑ worm burden  ↑­% of Mfpos mice ↑­ microfilaremia | (4) |
|  |  | IL5 | rIL5 | Eos | ­↑ parasite fecundity | (5) |
|  |  | Eotaxin | Eotaxin^-/-^  mice | Eos | ­↑ survival of adults | (6) |
|  |  | Histamine | HR1i | Eos | ↑ worm burden | (7) |
|  |  | IFNγ | IFNγ^-/-^  mice | Mac/Neu | ­↑ survival of adults ↑­microfilaremia | (8) |
|  |  | IFNγ/IL5 | IFNγ^-/-^ /IL5^-/-^  mice | Mac/Eos/Neu | ­↑ survival of adults ↑­microfilaremia | (3) |
|  |  | TLR4 | TLR4^-/-^  mice | Mac | ­↑ % of Mfpos mice | (9, 10) |
|  |  | IL33 | IL33R^-/-^ mice | Mac | ­↑ microfilaremia | (11) |
|  |  | B-cells | B-cell-deficient mice (μMT mice) | B-cells | No patent phase | (12) |
|  |  | B1-cells | B1-cell-deficient mice (Xid) | B1-cells | ­↑ Worm burden  ↑­ microfilaremia | (13) |
|  |  | Treg | Treg depletion (α-CD25 and α-GITR ab) | Tregs | ↓ worm burden | (14) |
| C57BL/6 | Semi-resistant (adults killed before patency) | lymphocytes | Rag2IL-2Rγ^-/-^  mice | T, B and NK cells | ­ ↑ survival of adults  development of patent phase | (15) |
|  |  | IL4 | IL4^-/-^  mice | Mac | ↑ survival of adults  development of patent phase | (16) |
|  |  | NOD2 | NOD2^-/-^  mice | Neu | Parasites eliminated before patency | (17) |
| 129/SvJ | Semi-resistant  (adults killed before patency) | EPO | EPO^-/-^  mice | Eos | Parasites eliminated before patency | (18) |
|  |  | MBP | MBP^-/-^  mice | Eos | Parasites eliminated before patency | (18) |
| FVB | Semi-resistant (adults killed before patency) | IL10 | IL-10 overexpressing macrophages  (macIL-10tg) | Mac | ↑survival of adults  development of patent phase | (19) |
| CBA/Ca | Semi-resistant  (adults survive >60 days but no patency) | IL5 | IL5 overexpressing mice | Eos | ↓ worm burden  No patent phase | (20) |

1. Volkmann L, Saeftel M, Bain O, Fischer K, Fleischer B, Hoerauf A. Interleukin-4 is essential for the control of microfilariae in murine infection with the filaria Litomosoides sigmodontis. Infection and immunity. 2001;69(5):2950-6. Epub 2001/04/09.

2. Volkmann L, Bain O, Saeftel M, Specht S, Fischer K, Brombacher F, et al. Murine filariasis: interleukin 4 and interleukin 5 lead to containment of different worm developmental stages. Med Microbiol Immunol. 2003;192(1):23-31. Epub 2003/02/20.

3. Saeftel M, Arndt M, Specht S, Volkmann L, Hoerauf A. Synergism of gamma interferon and interleukin-5 in the control of murine filariasis. Infection and immunity. 2003;71(12):6978-85. Epub 2003/11/26.

4. Ritter M, Tamadaho RS, Feid J, Vogel W, Wiszniewsky K, Perner S, et al. IL-4/5 signalling plays an important role during Litomosoides sigmodontis infection, influencing both immune system regulation and tissue pathology in the thoracic cavity. Int J Parasitol. 2017;47(14):951-60. Epub 2017/09/02.

5. Babayan SA, Read AF, Lawrence RA, Bain O, Allen JE. Filarial parasites develop faster and reproduce earlier in response to host immune effectors that determine filarial life expectancy. PLoS Biol. 2010;8(10):e1000525. Epub 2010/10/27.

6. Gentil K, Lentz CS, Rai R, Muhsin M, Kamath AD, Mutluer O, et al. Eotaxin-1 is involved in parasite clearance during chronic filarial infection. Parasite Immunol. 2014;36(2):60-77. Epub 2013/10/12.

7. Fox EM, Morris CP, Hubner MP, Mitre E. Histamine 1 Receptor Blockade Enhances Eosinophil-Mediated Clearance of Adult Filarial Worms. PLoS Negl Trop Dis. 2015;9(7):e0003932. Epub 2015/07/24.

8. Saeftel M, Volkmann L, Korten S, Brattig N, Al-Qaoud K, Fleischer B, et al. Lack of interferon-gamma confers impaired neutrophil granulocyte function and imparts prolonged survival of adult filarial worms in murine filariasis. Microbes Infect. 2001;3(3):203-13. Epub 2001/05/19.

9. Rodrigo MB, Schulz S, Krupp V, Ritter M, Wiszniewsky K, Arndts K, et al. Patency of Litomosoides sigmodontis infection depends on Toll-like receptor 4 whereas Toll-like receptor 2 signalling influences filarial-specific CD4(+) T-cell responses. Immunology. 2016;147(4):429-42. Epub 2015/12/31.

10. Pfarr KM, Fischer K, Hoerauf A. Involvement of Toll-like receptor 4 in the embryogenesis of the rodent filaria Litomosoides sigmodontis. Med Microbiol Immunol. 2003;192(1):53-6. Epub 2003/02/20.

11. Ajendra J, Specht S, Neumann AL, Gondorf F, Schmidt D, Gentil K, et al. ST2 deficiency does not impair type 2 immune responses during chronic filarial infection but leads to an increased microfilaremia due to an impaired splenic microfilarial clearance. PLoS One. 2014;9(3):e93072. Epub 2014/03/26.

12. Martin C, Saeftel M, Vuong PN, Babayan S, Fischer K, Bain O, et al. B-cell deficiency suppresses vaccine-induced protection against murine filariasis but does not increase the recovery rate for primary infection. Infection and immunity. 2001;69(11):7067-73. Epub 2001/10/13.

13. Al-Qaoud KM, Fleischer B, Hoerauf A. The Xid defect imparts susceptibility to experimental murine filariosis--association with a lack of antibody and IL-10 production by B cells in response to phosphorylcholine. Int Immunol. 1998;10(1):17-25. Epub 1998/03/06.

14. Taylor MD, LeGoff L, Harris A, Malone E, Allen JE, Maizels RM. Removal of regulatory T cell activity reverses hyporesponsiveness and leads to filarial parasite clearance in vivo. J Immunol. 2005;174(8):4924-33. Epub 2005/04/09.

15. Layland LE, Ajendra J, Ritter M, Wiszniewsky A, Hoerauf A, Hubner MP. Development of patent Litomosoides sigmodontis infections in semi-susceptible C57BL/6 mice in the absence of adaptive immune responses. Parasites & vectors. 2015;8:396. Epub 2015/07/26.

16. Le Goff L, Lamb TJ, Graham AL, Harcus Y, Allen JE. IL-4 is required to prevent filarial nematode development in resistant but not susceptible strains of mice. Int J Parasitol. 2002;32(10):1277-84. Epub 2002/09/03.

17. Ajendra J, Specht S, Ziewer S, Schiefer A, Pfarr K, Parcina M, et al. NOD2 dependent neutrophil recruitment is required for early protective immune responses against infectious Litomosoides sigmodontis L3 larvae. Sci Rep. 2016;6:39648. Epub 2016/12/23.

18. Specht S, Saeftel M, Arndt M, Endl E, Dubben B, Lee NA, et al. Lack of eosinophil peroxidase or major basic protein impairs defense against murine filarial infection. Infection and immunity. 2006;74(9):5236-43. Epub 2006/08/24.

19. Specht S, Taylor MD, Hoeve MA, Allen JE, Lang R, Hoerauf A. Over expression of IL-10 by macrophages overcomes resistance to murine filariasis. Exp Parasitol. 2012;132(1):90-6. Epub 2011/10/01.

20. Martin C, Le Goff L, Ungeheuer MN, Vuong PN, Bain O. Drastic reduction of a filarial infection in eosinophilic interleukin-5 transgenic mice. Infection and immunity. 2000;68(6):3651-6. Epub 2000/05/19.
